# How Host Phylogeny, Diet, and Habitat Affect Gut Microbial Diversity in Wild Snakes

**DOI:** 10.1002/ece3.73902

**Published:** 2026-07-01

**Authors:** Jiaqi Zhang, Chunmei Fu, Songwen Tan, Bing Lyu, Guocheng Shu, Lei Shi, Yayong Wu, Peng Guo

**Affiliations:** ^1^ Faculty of Agriculture, Forestry and Food Engineering Yibin University Yibin China; ^2^ Yibin Key Laboratory of Zoological Diversity and Ecological Conservation & Yibin Inspection and Quarantine Engineering Technology Research Center of Animal and Plant Yibin University Yibin China; ^3^ College of Life Sciences Xinjiang Agricultural University Urumqi China; ^4^ College of Life Sciences Shenyang Normal University Shenyang China; ^5^ School of Ecology and Environment Tibet University Lhasa China

**Keywords:** diet, gut microbiota, habitat, metagenomic, phylogeny, wild snakes

## Abstract

Gut microbiota plays critical roles in host digestion, immune regulation, neurochemical signaling, and metabolic homeostasis. Based on wild snakes (73 individuals from 23 species) from China, we explored the composition, characteristics, and functions of gut microbes across different groups using fecal metagenomic samples; further we explored the relative contributions of host phylogeny, diet, and habitat to the microbial structure. Among 23 wild snake species, the dominant gut microbial phyla were Proteobacteria, Bacteroidetes, Firmicutes, and Actinobacteria, with *Bacteroides*, *Salmonella*, *Citrobacter*, and *Aeromonas* comprising the major genera. Mantel test revealed a significant correlation (*r* = 0.3173, *p* = 0.0055) between microbial composition at the genus level and host genetic divergence (*p*‐distance), indicating potential phylogenetic influence on gut microbial profiles. While α‐diversity and principal coordinate analysis showed no marked differences across different subgroups. Linear discriminant analysis effect size demonstrated notable differences in the gut microbes of the terrestrial snakes with different diets and vertebrate‐feeding snakes with different habitats. Functional annotation of microbial genes indicated enrichment in metabolic processes, as well as environmental and genetic information processing. Carbohydrate‐active enzymes were predominantly from GT2, GT4, GT51, and GH23 families. Linear discriminant analysis effect size showed different diets and habitats had distinct differential taxa. Additionally, antibiotic resistance gene profiles varied across groups, with *acrB*, *AcrF*, *MexB*, *acrD*, and *mdtF* being most prevalent. Future studies should increase the samples and comprehensively consider different ecological factors to explore the impacts on the composition and functions of snake gut microbes on different evolutionary, which will provide a deeper understanding of the interrelationships between snake gut microbes and their hosts.

## Graphical abstract

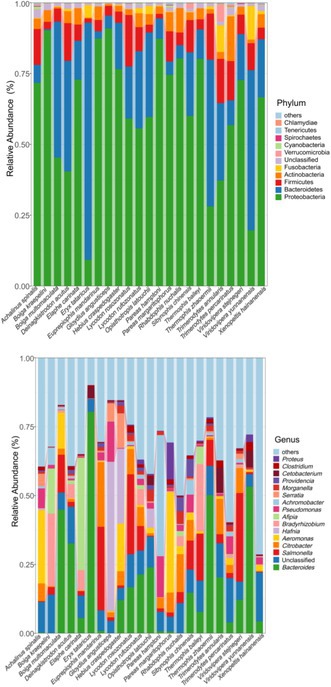

## 1 Introduction

Gut microbiota represent a dynamic and complex system within the intestinal tract, contributing to host physiology through diverse mechanisms. These include promoting digestion and nutrient absorption (Zhang et al. 2022; Wang et al. 2023), synthesizing essential bioactive compounds, and regulating neurobehavioral processes (Yachida et al. 2019; Siddiqui et al. 2022; Yang et al. 2024). Accumulating evidence across vertebrates suggests that gut microbial communities are shaped by a complex interplay among host phylogenetic history, dietary composition, and habitat characteristics (Brown et al. 2023; Rojas et al. 2021; Zhang et al. 2024). The concept of “phylosymbiosis” captures the evolutionary congruence between host lineages and their associated microbiomes (Brucker and Bordenstein 2013), wherein phylogenetically closer species tend to exhibit greater microbial similarity compared to more distantly related taxa (Youngblut et al. 2019). Habitat and diet often lead to significant differences in composition and diversity of the gut microbes in different taxonomic groups or in different populations of the same species (Zhu et al. 2022; Wu 2024).

Previous studies have shown that “phylosymbiosis” could be observed between hosts with the same habitat or diet in one type of phylogenetic relationship, as well as between hosts with different habitats or diets (Amato et al. 2019; Kartzinel et al. 2019; Knowles et al. 2019). Comparative studies of African herbivores have revealed that gut microbiota are often highly species‐specific and exhibit phylosymbiotic patterns, with closely related hosts harboring more similar microbial communities. However, in bovine species, microbial composition appears to be more strongly shaped by dietary inputs than by phylogenetic relatedness (Rojas et al. 2021), suggesting that ecological factors can override evolutionary signals at finer taxonomic scales. Similar patterns have been observed in sympatric small mammals from African grasslands, including closely related mice and hares, where gut microbial dissimilarity increases with host phylogenetic distance. However, across the broader rodent assemblage, microbial diversity and composition correlate more strongly with ecological factors such as body size, dietary breadth, and trophic overlap than with phylogenetic relatedness (Brown et al. 2023).

Functionally, the gut microbiota profoundly influences host metabolic homeostasis (Gao et al. 2024), immune function (Wei et al. 2023), and physiological adaptation to environmental challenges (Li, Liang, et al. 2020; Li, Peng, et al. 2020). These microbial communities regulate the intestinal environment, enhance nutrient absorption and utilization, and contribute to immunomodulation and metabolic regulation, all of which are critical for overall animal health (Zhang et al. 2020). Functional gene annotation has shown that gut microbes in animals are primarily associated with nutrient metabolism, immune processes, and information processing (Li et al. 2021). Among these, carbohydrate metabolism constitutes a dominant component of gut microbial function across vertebrate species (Zhou et al. 2019; Liu, Guo, et al. 2021; Liu, Yang, et al. 2021; Jiang et al. 2023). In particular, analyses of humans, rhesus macaques, and dogs inhabiting various altitudinal environments have shown that carbohydrate metabolism is the most enriched Kyoto Encyclopedia of Genes and Genomes (KEGG) second‐tier pathway (Dong 2020). However, the specific contributions of carbohydrate‐active enzymes (CAZymes) to these metabolic processes in animals remain insufficiently characterized.

In parallel, wild vertebrates frequently harbor conditionally pathogenic and drug‐resistant bacteria, posing potential risks for both host health and environmental transmission (Tang, Wang, et al. 2019; Tang, Yang, et al. 2019; Tang, Zhu, et al. 2019; Zhang et al. 2024). Microbiome studies in reptiles, including snakes and turtles, have identified widespread antibiotic‐resistant strains, often with conserved susceptibility profiles across species (Cui et al. 2025). Therefore, detection and study of antibiotic resistance genes (ARGs) will be beneficial to understand what antibiotics are acting in animals, and to hypothesize which conditionally pathogenic and resistant bacteria produce these antibiotics and what roles these ARGs play.

This study investigated the taxonomic and functional composition of gut microbiota across 23 wild snake species through metagenomic next‐generation sequencing (mNGS), integrated with phylogenetic frameworks derived from molecular data. Microbial community variation was analyzed in relation to host phylogeny, dietary strategies, and habitat types at both species‐ and family‐level scales. Functional gene annotation was performed using the kOfam database, while CAZymes and ARGs were systematically characterized using the Carbohydrate‐Active enZYmes Database (CAZy, https://www.cazy.org/) and Comprehensive Antibiotic Resistance Database (CARD, https://card.mcmaster.ca/) to predict their compositional diversity and potential biological roles. We predicted that (1) host phylogeny would be the primary driver of gut microbial composition due to phylosymbiosis; (2) diet would exert a stronger influence than habitat at finer taxonomic scales; and (3) the main functional information is associated with metabolism and environmental, genetic information processing, functional profiles (CAZymes, ARGs) would differ between dietary groups.

## 2 Materials and Methods

### 2.1 Sample Collection and DNA Extraction

**FIGURE 1:**
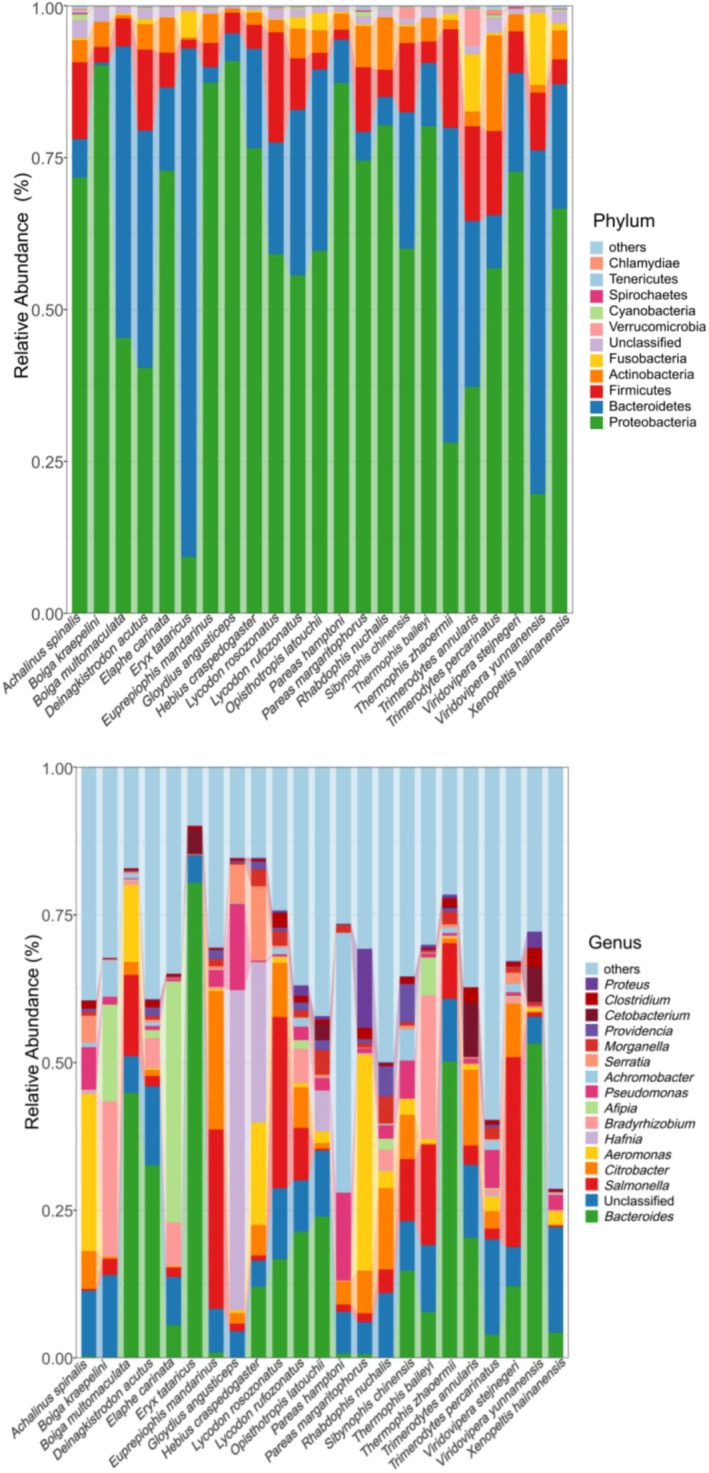
Relative abundance of gut bacteria in 23 snake species at the phylum (up) and genus (down) levels. *X*‐axis indicates species, and different colors represent bacterial phylum and genus.

A total of 73 adult snakes representing 23 species, 17 genera, and nine families were collected across China between July 2021 and September 2023. All individuals were healthy and classified into two dietary groups (invertebrate‐ and vertebrate‐feeding, Appendix S4) and four habitat types (arboreal, fossorial, semiaquatic, and terrestrial) based on published data (Zhao 2006; Liu et al. 2012; Wang, Che, et al. 2020; Wang, Ren, et al. 2020; Guo et al. 2022, Guo and Che 2024; Harrington et al. 2018; Huang et al. 2022) (Figure 1). More sample collection and DNA extraction details were performed as previously described (Zhang et al. 2024). In brief, Fecal samples were transferred into 2 mL tubes preloaded with 50% glycerol (Fuyu Fine Chemical Co. Ltd., Tianjin, China), snap‐frozen in liquid nitrogen, and subsequently stored at −80°C. And genomic DNA was extracted from fecal material by Novogene (Beijing, China) using a Rapid Plus DNA Library Prep Kit for Illumina (RK20208; Shanghai Molecular Research and Development Center, Shanghai, China). All snakes were released at their original collection sites following sampling. Collection sites ranged in elevation from 128 to 4039 m. Detailed species information was provided in Appendix S1.

### 2.2 Library Construction, Quality Control, and Sequencing

These steps were performed as previously described (Zhang et al. 2024). Approximately 0.2 μg of DNA per sample was used as input material for the DNA library preparations. The genomic DNA samples were fragmented by sonication to a size of 350 bp, then polished, A‐tailed, and ligated with the full‐length adapter for Illumina sequencing, followed by polymerase chain reaction (PCR) amplification. The resulting PCR products were purified, library quality (by Agilent 5400 system) and quantified (by quantitative PCR, 1.5 nM). The qualified libraries, each with an effective concentration over 2 nM, were pooled and sequenced on the Illumina HiSeq 2500/MiSeq platform using a paired‐end 150 bp (PE150) strategy. The original fluorescence image files obtained were transformed to short reads (raw reads) by base calling and BclToFastq, and recorded in FASTQ format (Cock et al. 2010).

### 2.3 Metagenomic Data Processing and Analysis

Quality assessment criteria for raw reads and part of the metagenomic data analysis were performed as previously described (Zhang et al. 2024). In brief, the read pairs discarded if adapter contamination was present, more than 10% of bases were uncertain, and the proportion of low‐quality (Phred quality < 5) bases exceeded 50% in either read by Fastp v0.23.1 (Chen et al. 2018). And Sequence alignment against host genomes was performed using Bowtie2 v2.4.1 (Langmead and Salzberg 2012; Langmead et al. 2019). The sequences were aligned to standard databases using Kraken2 v2.0.7 to obtain annotation information and abundance tables (Wood et al. 2019). Target sequences were assembled using Megahit v1.2.9 (Li et al. 2016) with parameters: ‐t 8 ‐m 0.95 ‐‐min‐contig‐len 300 ‐‐k‐min 51 ‐‐k‐max 127 ‐‐k‐step 20. Assembled sequences were clustered using Cd‐hit v4.8.1 (Fu et al. 2012) with parameters: ‐c 0.95 ‐aS 0.9 ‐g 1 ‐sc 1 ‐sf 1 ‐T 8 ‐M 8000. Protein‐coding sequences were predicted using Prodigal v2.6.3 (Hyatt et al. 2010). Quantification was performed using Salmon v0.14.1 (Patro et al. 2017). The only modifications in this study were the reference genomes for host species were downloaded from the National Center for Biotechnology Information (NCBI) database (https://www.ncbi.nlm.nih.gov/) and used to filter host‐derived sequences (Appendix S2). Functional annotation of predicted proteins was performed using KofamScan against the KOfam database to obtain KEGG Ortholog assignments, followed by visualization in R. Functional categorization of CAZymes was conducted using CAZy v3.0.5 (Lombard et al. 2014) with Run_dbcan (Zhang, Li, et al. 2018; Zhang, Yohe, et al. 2018) and CARD v4.0.0 (Alcock et al. 2023) with Diamond v2.1.10 (Buchfink et al. 2015).

The correlation between host phylogeny (*p*‐distance matrix calculated based on COI) and gut microbial composition (relative abundance matrix) was assessed using Spearman rank correlation (Spearman 2010) and Mantel tests (Mantel 1967) implemented in the vegan package in R v23.3.0 (R Development Core Team 2020). Visualizations, including Circos plots, clustered heatmaps, stacked linked histograms, and grid heatmaps, were generated using Wekemo Bioincloud (https://www.bioincloud.tech, Gao et al. 2024). Pie charts and petal diagrams were created with Chiplot (https://www.chiplot.online/). Percentage‐stacked column charts representing species abundance was calculated in R v4.3.1. Diversity indices included abundance‐based coverage estimation, Simpson's diversity index, Chao1 estimator, Shannon‐Weiner index, observed species count, and Goods's coverage index. Snakes were grouped by phylogeny, dietary category, and habitat type for comparative analyses (see below). Snakes were grouped by phylogeny, dietary category, and habitat type for comparative analyses (see below). Kruskal–Wallis and *t*‐tests were used to evaluate differences in α‐diversity among groups. Principal coordinate analysis (PCoA) based on Bray‐Curtis distance was performed to detect compositional variation in gut microbiota. Permutational multivariate analysis of variance (PERMANOVA) was used to assess group‐level differences in community structure using R v4.3.1. Additionally, linear discriminant analysis (LDA) effect size (LEfSe) was used to identify significant abundance difference components of wild snake gut bacteria from phylum to genus at different subgroups and for significant abundance difference components of CAZymes and ARGs of wild snake gut microbes at different diets and habitats. LDA was then applied to assess the impact of each component on differences in abundance (Segata et al. 2011), with the results visualized using R v4.3.1. The LDA criterion was set to a log‐transformed value greater than or equal to 2 with a base of 10.

### 2.4 Phylogenetic Relationship Construction

Four fragments of mitochondrial DNA, including cytochrome *c* oxidase subunit I (COI), cytochrome *b* (CYTB), NADH dehydrogenase subunit 2 (ND2) and NADH dehydrogenase subunit 4 (ND4), were retrieved from NCBI for all 23 focal snake species. *Argyrophis diardii* (Schlegel 1839) and *Indotyphlops braminus* (Daudin 1803) were selected as outgroups (Appendix S2) based on prior phylogenetic studies (Li, Liang, et al. 2020; Li, Peng, et al. 2020). Sequence alignment was performed using the Clustal *W* algorithm in MEGA v7.0 (Kumar et al. 2016), followed by manual correction to generate a concatenated dataset. The optimal partitioning scheme and substitution models were determined using PartitionFinder v2.2.1 (Lanfear et al. 2017). A Bayesian Inference (BI) tree was constructed using MrBayes v3.2.4 (Ronquist et al. 2012), with the resulting tree visualized using FigTree v1.4.4 (https://tree.bio.ed.ac.uk/software/figtree/). Pairwise uncorrected genetic distances (*p*‐distance) between species were calculated based on COI sequences (Wu, Hou, et al. 2023; Wu, Peng, et al. 2023).

## 3 Results

### 3.1 Metagenomic Next‐Generation Sequencing and Quality Assessment

A total of 3,307,404,304 sequences were obtained from the 73 samples of 23 snakes (invertebrate: 444,485,998 vs. vertebrate: 2,790,659,028; arboreal: 619,562,814 vs. terrestrial: 1,752,776,534 vs. fossorial: 192,272,156 vs. semiaquatic: 742,792,800). After quality control filtering, 2,919,205,564 high‐quality sequences were retained (invertebrate: 439,057,024 vs. vertebrate: 2,408,470,088; arboreal: 611,344,110 vs. terrestrial: 1,380,974,452 vs. fossorial: 189,976,402 vs. semiaquatic: 736,910,600). The results of the metagenomic next‐generation sequencing data of the obtained samples were excellent, indicating that most sequences were annotated and sampled samples were sufficient and representative for subsequent analyses (Table 1). Sequence length, TPM (transcripts per kilobase of exon model per million mapped reads) values, and number of reads were detailed in the Table 2.

**TABLE 1:** Quality of metagenomic next‐generation sequencing data across different dietary and habitat groups of 23 snake species (average values).

Classification		Q20 (%)	Q30 (%)	GC content (%)	Effective rate (%)
Diet	Invertebrate	97.37	93.25	47.57	98.73
	Vertebrate	97.07	92.39	46.74	98.98
Habitat	Arboreal	97.28	92.82	47.81	98.72
	Fossorial	97.51	93.37	43.64	98.80
	Semiaquatic	97.24	92.79	46.71	99.05
	Terrestrial	96.96	92.21	46.86	99.04

*Note:* Q20 and Q30: percentage of bases with Phred values > 20 and > 30, respectively, against total bases from Illumina HiSeq 2500/MiSeq; GC Content: percentage of G and C bases against total number of bases; Effective rate: ratio of valid data retained after filtering original data. The 23 snake species analyzed were categorized into two dietary groups (invertebrate‐ and vertebrate‐feeding), except *Xenopeltis hainanensis* , and four habitat types (arboreal, fossorial, semiaquatic, and terrestrial).

**TABLE 2:** Salmon‐based quantification of gut microbiota across dietary and habitat groups of 23 snake species (average values).

Classification		Length (bp)	Effective length (bp)	TPM	No. reads
Diet	Invertebrate	379.92	185.05	4.93	31.86
	Vertebrate	455.01	235.22	6.94	61.71
Habitat	Arboreal	496.56	266.56	9.56	84.83
	Fossorial	387.86	193.13	4.71	71.41
	Semiaquatic	440.58	227.73	5.85	62.49
	Terrestrial	423.66	211.53	5.61	37.85

*Note:* Length (bp): actual length of original FASTQ sequence; Effective length (bp): FASTQ sequence effective length; TPM: transcripts per thousand base transcripts per million mapped reads, summarizing length, expression, and number of genes, used to estimate sample expression; Num reads: estimated number of reads matched to each sample. The 23 snake species analyzed were categorized into two dietary groups (invertebrate‐ and vertebrate‐feeding, except *Xenopeltis hainanensis*) and four habitat types (arboreal, fossorial, semiaquatic, and terrestrial).

### 3.2 Composition and Characteristics of Gut Microbiota in Wild Snakes

**FIGURE 2:**
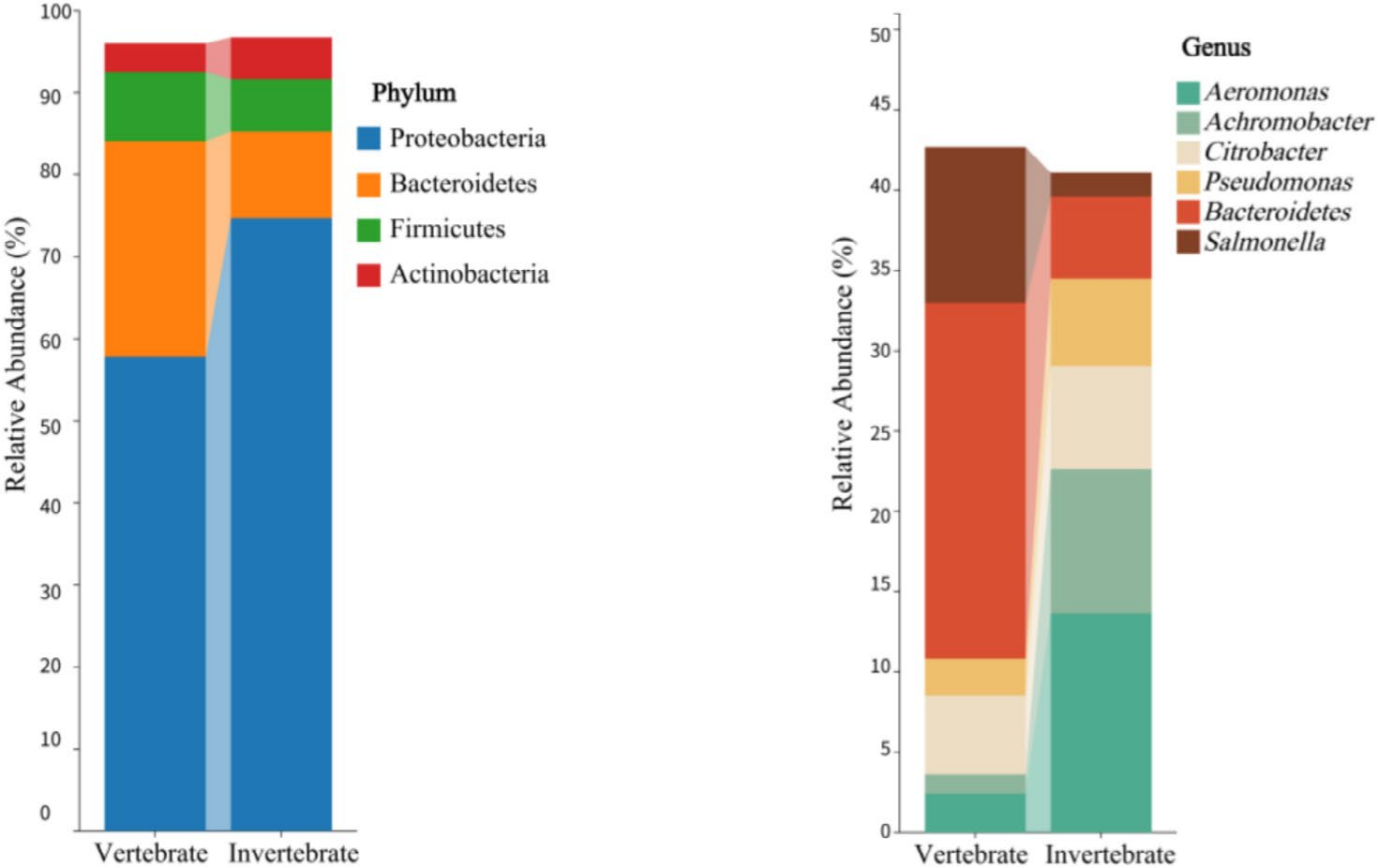
Dominant phyla (left) and genera (right) identified in 23 wild snake species across different dietary types. *X*‐axis indicates different dietary types, and color represents different bacterial phyla (relative abundance ≥ 3%) and genera (relative abundance ≥ 5%).

**FIGURE 3:**
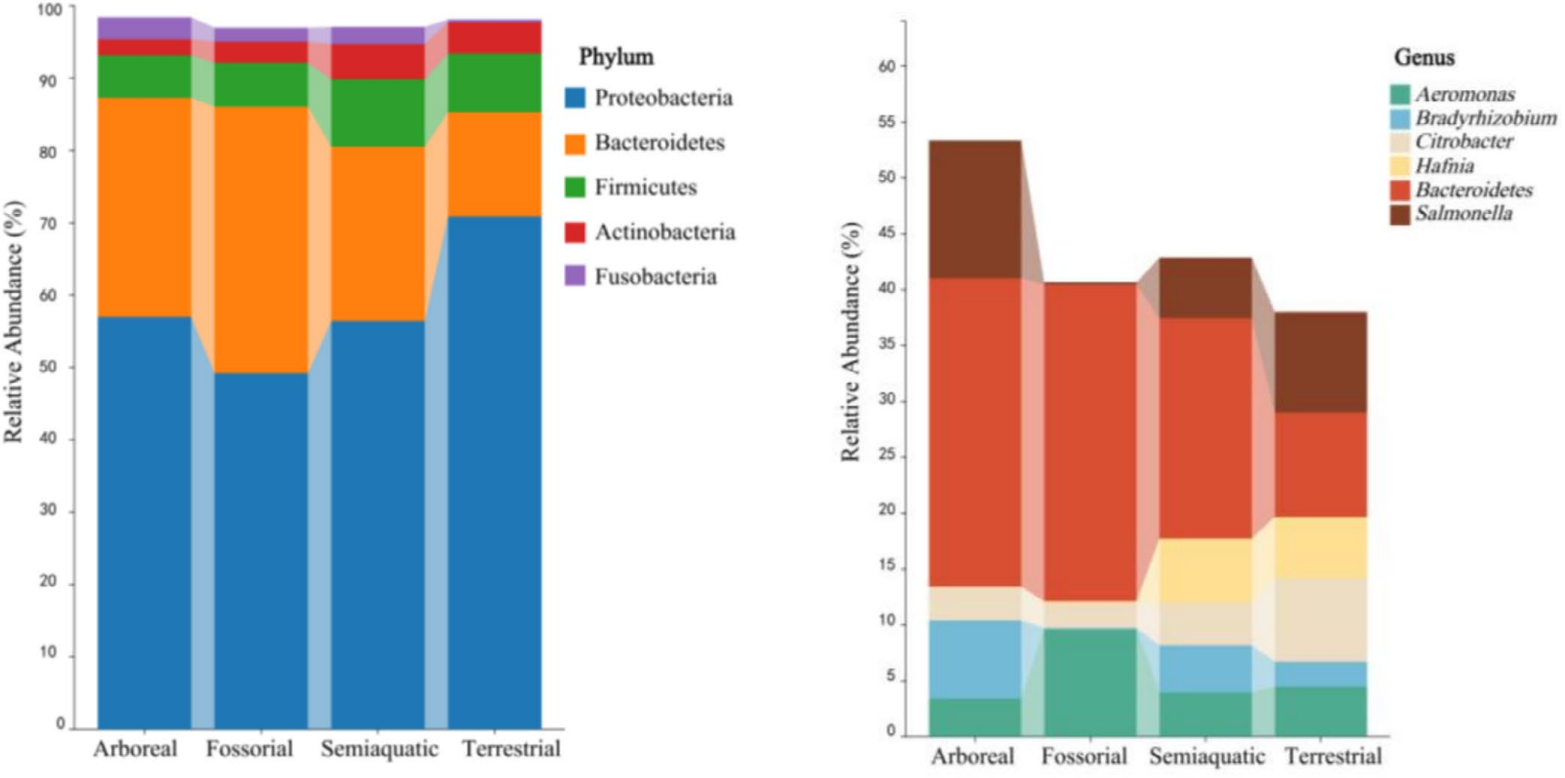
Dominant phyla (left) and genera (right) identified in 23 wild snake species across different habitat types. *X*‐axis indicates different habitats types, and color represents different bacterial phyla (relative abundance ≥ 3%) and genera (relative abundance ≥ 5%).

Based on metagenomic sequencing, the gut microbiota of the 23 wild snake species was predominantly composed of Bacteria (93.79%), Eukaryota (5.82%), Viruses (0.30%), and Archaea (0.11%). A total of 20,341 bacterial species were identified, belonging to 49 phyla, 82 classes, 187 orders, 439 families, and 2428 genera. The most abundant phyla (relative abundance ≥ 3%) were Proteobacteria (61.83% ± 22.31%), Bacteroidetes (22.63% ± 20.46%), Firmicutes (7.77% ± 5.07%), and Actinobacteria (3.92% ± 3.21%) (Figure 2), while the most abundant genera (relative abundance ≥ 4%) were *Bacteroides* (17.69% ± 20.91%), *Salmonella* (7.50% ± 10.01%), *Citrobacter* (5.05% ± 5.67%), and *Aeromonas* (4.81% ± 9.35%) (Figure 1). The dominant phyla and genera observed across the two dietary groups and four habitat types were shown in Figures 2 and 3.

### 3.3 Gut Microbiota Differences in Subgroups of Wild Snakes

**FIGURE 4:**
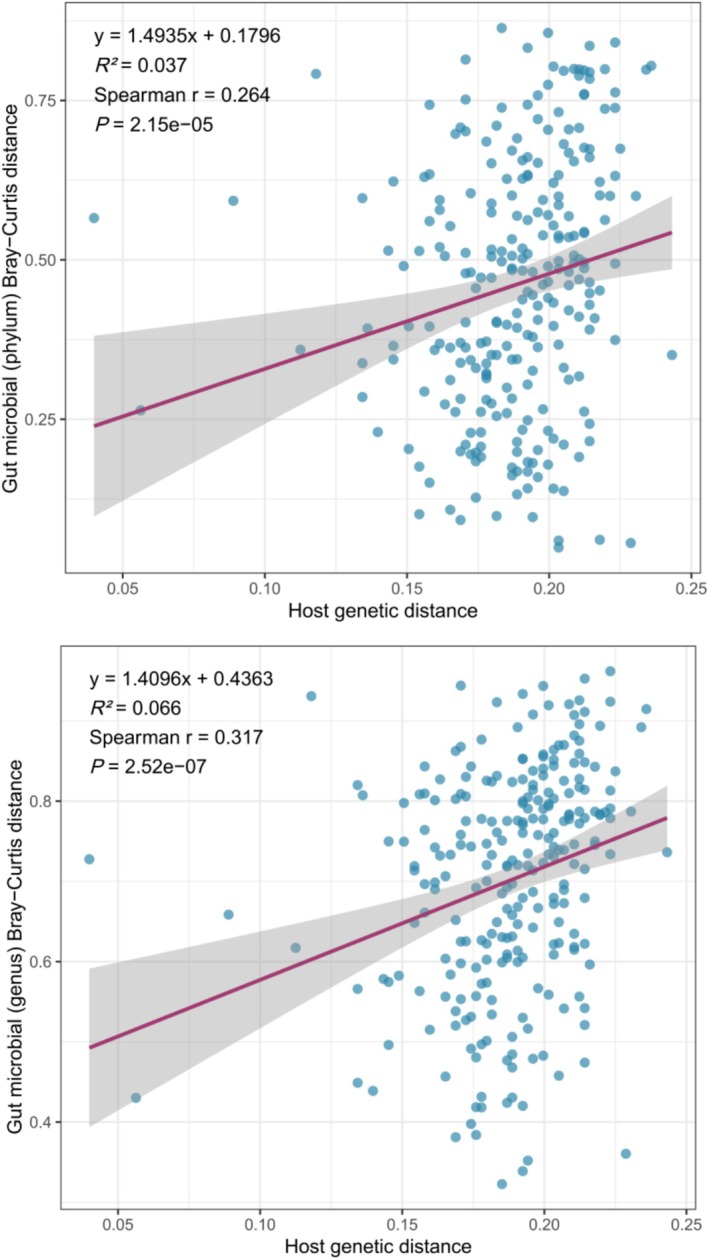
Correlation between host genetic distance (based on COI) and gut microbial Bray‐Curtis distance. Linear regression analysis was used to fit the relationship, and Mantel test with 9999 permutations was performed based on Spearman correlation. *R* ^2^ represents the goodness of fit of linear regression, *r* and *p* indicate the correlation coefficient and significance level of Mantel test.

**FIGURE 5:**
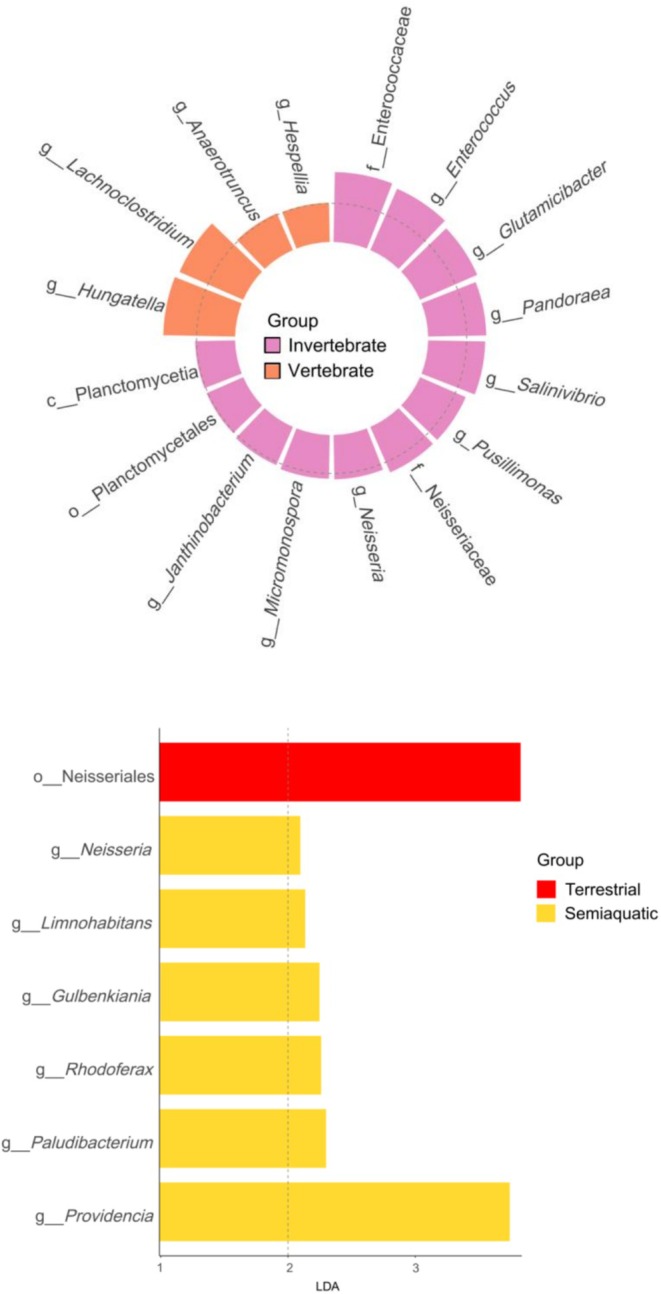
LEfSe analysis of differentially abundant taxa across snake subgroups. LDA scores indicate taxa with significantly different relative abundances in subgroups I (invertebrate vs. vertebrate) and II (semiaquatic vs. terrestrial). Bar length indicates degree of influence of a taxon. Only taxa meeting a significant LDA threshold ≥ 2 are shown. The dashed line represents LDA threshold = 2, and the circular column chart indicates the LDA score in the left figure. Letters p, c, o, f, and g represent phylum, class, order, family, and genus, respectively.

Mantel test results indicated no significant correlation between gut microbiota and host phylogeny (Appendix S3) at the phylum level (*r* = 0.2637, *p* = 0.0163), and a significant correlation was observed at the genus level (*r* = 0.3173, *p* = 0.0055) (Figure 4). α‐diversity analyses revealed no significant differences among the four snake subgroups (I–IV) (Table 3). The *R* ^2^ values were generally low (0.07–0.37) and all *p*‐values are > 0.05 by the PCoA, indicating host phylogeny, diet, and habitat did not have a significant overall impact on gut microbial structure (Table 4). LEfSe analysis revealed significant differences in microbial abundance across dietary and habitat subgroups. In subgroup I, 12 differential microbial taxa were enriched in invertebrate‐feeding species, primarily from Actinobacteria, Firmicutes, Proteobacteria, and Planctomycetes, while four taxa from Firmicutes were enriched in vertebrate‐feeding species (Figure 5). In subgroup II, semiaquatic species harbored six distinct microbial taxa and terrestrial species harbored one, all belonging to Betaproteobacteria and Gammaproteobacteria within the phylum Proteobacteria (Figure 5, Table 3).

**TABLE 3:** Gut microbial α‐diversity of different subgroups in snakes.

Subgroups				ACE	Simpson	Chao1
	Classification	Habitat	Diet			
I	Serpentes (five families)	Terrestrial	Invertebrate	3.98 ± 0.25	0.94 ± 0.01	19,731.35 ± 807.47
			Vertebrate	3.92 ± 0.43	0.94 ± 0.02	19,836.68 ± 941.55
II	Serpentes (six families)	Arboreal	Vertebrate	3.60 ± 0.16	0.93 ± 0.01	18,821.51 ± 1437.64
		Semiaquatic		4.91 ± 4.10	0.96 ± 0.95	22,318.85 ± 20,274.50
		Terrestrial		3.92 ± 0.43	0.94 ± 0.02	20,497.35 ± 750.51
III	Colubridae	Terrestrial	Vertebrate	3.89 ± 0.27	0.94 ± 0.01	21,079.54 ± 417.39
		Arboreal		3.54 ± 0.20	0.93 ± 0.01	18,378.42 ± 1072.15
IV	Colubridae	Arboreal	Vertebrate	3.54 ± 0.20	0.93 ± 0.01	18,253.87 ± 1085.26
	Viperidae			3.65 ± 0.07	0.94 ± 0.00	20,322.78 ± 952.77
	Dipsadidae	Semiaquatic		3.81 ± 0.15	0.94 ± 0.00	19,165.40 ± 379.02
	Natricidae			4.29 ± 0.55	0.95 ± 0.01	20,999.07 ± 933.81
	Colubridae	Terrestrial		3.89 ± 0.27	0.94 ± 0.01	21,060.39 ± 451.07
	Viperidae			3.76 ± 0.62	0.93 ± 0.02	20,665.65 ± 1047.67

Subgroups				Shannon	Observed species	Goods's coverage
	Classification	Habitat	Diet			
I	Serpentes (five families)	Terrestrial	Invertebrate	19,551.59 ± 644.59	18,211.00 ± 148.94	1.00 ± 0.00
			Vertebrate	19,959.59 ± 760.55	18,200.71 ± 1518.68	1.00 ± 0.00
II	Serpentes (six families)	Arboreal	Vertebrate	18,617.72 ± 1370.59	17,297.25 ± 1685.59	1.00 ± 0.00
		Semiaquatic		22,114.91 ± 19,968.21	21,791.00 ± 18,976.60	1.00 ± 1.00
		Terrestrial		20,202.54 ± 674.43	19,218.29 ± 1200.62	1.00 ± 0.00
III	Colubridae	Terrestrial	Vertebrate	20,758.95 ± 405.96	20,134.50 ± 481.25	1.00 ± 0.00
		Arboreal		18,203.88 ± 1098.88	16,924.50 ± 1244.50	1.00 ± 0.00
IV	Colubridae	Arboreal	Vertebrate	18,097.55 ± 1144.12	17,037.00 ± 1247.00	1.00 ± 0.00
	Viperidae			20,008.40 ± 890.37	19,256.50 ± 939.50	1.00 ± 0.00
	Dipsadidae	Semiaquatic		18,809.60 ± 445.24	17,486.00 ± 984.00	1.00 ± 0.00
	Natricidae			20,726.53 ± 984.58	19,951.67 ± 1302.37	1.00 ± 0.00
	Colubridae	Terrestrial		20,751.58 ± 434.24	20,132.00 ± 502.16	1.00 ± 0.00
	Viperidae			20,381.39 ± 1024.15	19,210.00 ± 1583.00	1.00 ± 0.00

*Note:* All the data in the table were rounded to two decimal places (the values after rounding).

**TABLE 4:** Principal coordinate analysis (PCoA) of bacterial community diversity among 23 wild snake species across different subgroups, based on Bray‐Curtis distance matrices.

Subgroup	Classification	Habitat	Diet	Species	PERMANOVA
I	Serpentes (five families)	Terrestrial	Invertebrate vs. Vertebrate	*Pareas hamptoni* , *Pareas margaritophorus* , *Rhabdophis nuchalis* vs. *Deinagkistrodon acutus* , *Gloydius angusticeps*, *Elaphe carinata* , *Euprepiophis mandarinus* , *Lycodon rufozonatus* , *Lycodon rosozonatus* , *Sibynophis chinensis*	*R* ^2^ = 0.13, *F* = 1.15, *p* = 0.21
II	Serpentes (six families)	Terrestrial vs. Semiaquatic vs. Arboreal	Vertebrate	*Deinagkistrodon acutus* , *Gloydius angusticeps*, *Elaphe carinata* , *Euprepiophis mandarinus* , *Lycodon rufozonatus* , *Lycodon rosozonatus* , *Sibynophis chinensis* vs. *Trimerodytes annularis*, *Hebius craspedogaster* , *Trimerodytes percarinatus*, *Thermophis zhaoermii* , *Thermophis baileyi* vs. *Boiga kraepelini* , *Boiga multomaculata* , *Viridovipera stejnegeri*, *Viridovipera yunnanensis*	*R* ^2^ = 0.07, *F* = 0.51, *p* = 0.96
III	Colubridae	Terrestrial vs. Arboreal	Vertebrate	*Elaphe carinata* , *Euprepiophis mandarinus* , *Lycodon rufozonatus* , *Lycodon rosozonatus* vs. *Boiga kraepelini* , *Boiga multomaculata*	*R* ^2^ = 0.31, *F* = 1.78, *p* = 0.20
IV	Colubridae vs. Viperidae	Terrestrial	Vertebrate	*Elaphe carinata* , *Euprepiophis mandarinus* , *Lycodon rufozonatus* , *Lycodon rosozonatus* vs. *Deinagkistrodon acutus* , *Gloydius angusticeps*	*R* ^2^ = 0.21, *F* = 1.07, *p* = 0.47
	Colubridae vs. Viperidae	Arboreal	Vertebrate	*Boiga kraepelini* , *Boiga multomaculata* vs. *Viridovipera stejnegeri*, *Viridovipera yunnanensis*	*R* ^2^ = 0.37, *F* = 1.16, *p* = 0.33
	Natricidae vs. Dipsadidae	Semiaquatic	Vertebrate	*Trimerodytes annularis*, *Hebius craspedogaster* , *Trimerodytes percarinatus* vs. *Thermophis zhaoermii* , *Thermophis baileyi*	*R* ^2^ = 0.22, *F* = 0.84, *p* = 0.70

*Note:* In the PCOA analysis, the results are presented based on the differences detected by the PERMANOVA.

### 3.4 Functional Prediction of Gut Microbiota in Wild Snakes

**FIGURE 6:**
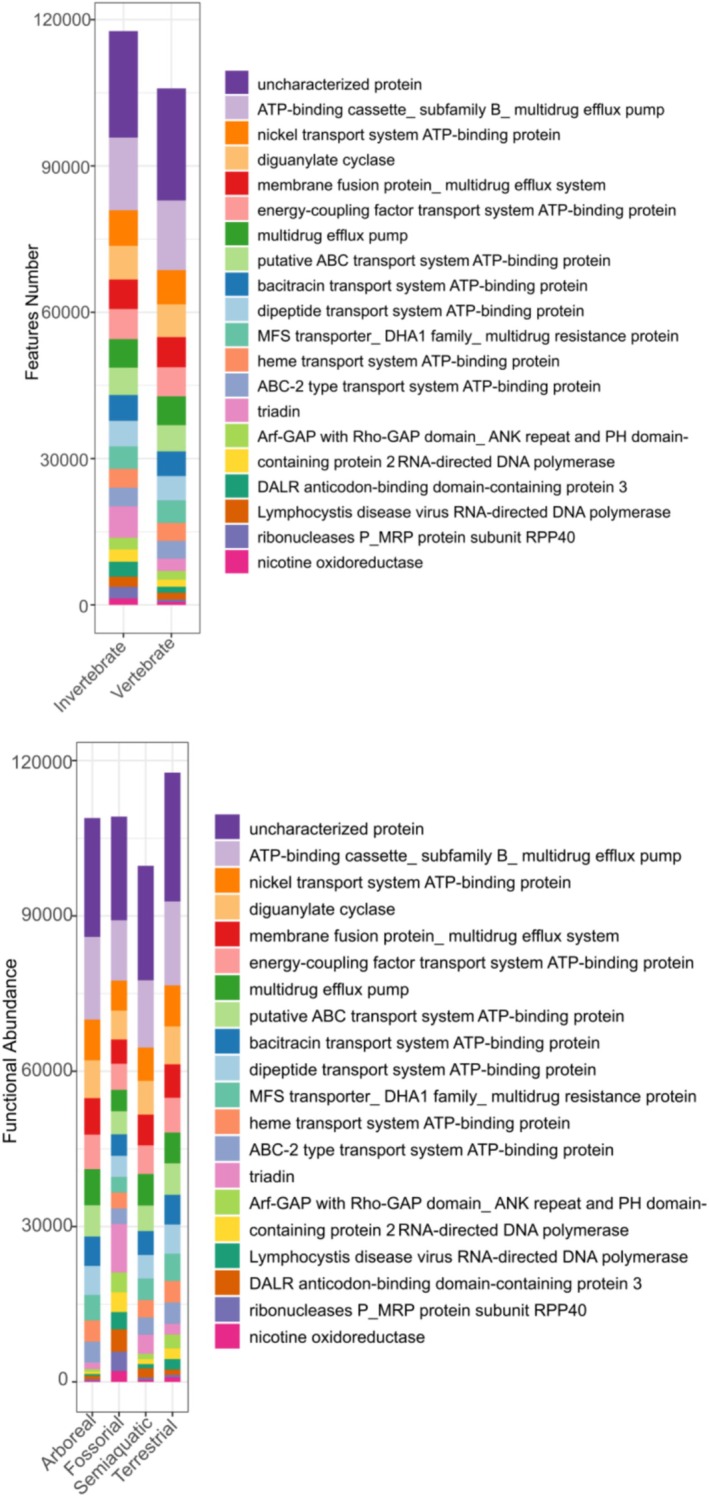
Functional gene abundance in gut microbiota of 23 wild snake species across dietary (up) and habitat (down) groups. *X*‐axis indicates different groups, and color represents different gene functions, and the *Y*‐axis represents the number of sequences corresponding to the function.

Based on functional annotation of gut microbial genes, the 10 most abundant predicted functions were shown in Figure 6 (from bottom to top). These functions were primarily associated with metabolic activity and the processing of environmental and genetic information. The main functions of the snakes that feed on invertebrates included encoding “triadin” genes, but did not include encoding “dipeptide transport system ATP‐binding protein” (among the top 10 in terms of function) genes at the dietary groups. The main functions of snakes living in burrows included encoding “trimerin” genes and “DALR anticodon‐binding domain‐containing protein 3”, but not “ATP‐binding protein of dipeptide transport system” and “MFS transporter, DHA1 family, multidrug resistance protein”(among the top 10 in terms of function) at different habitat groups.

**FIGURE 7:**
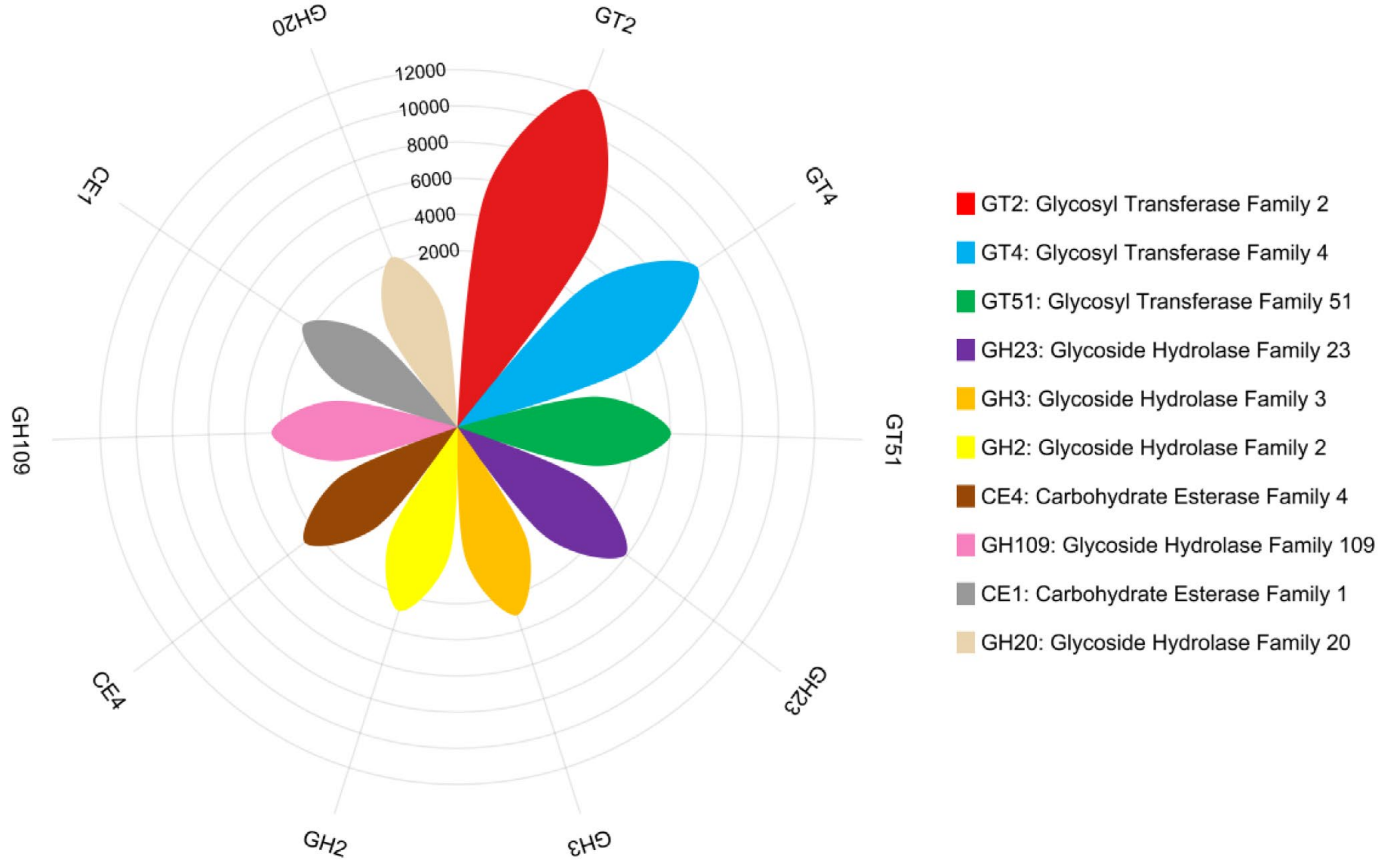
Abundance of the top 10 CAZymes in 23 wild snake species. The numbers represent the sequence counts of the corresponding carbohydrate enzyme families, and color represents different families of enzymes.

**FIGURE 8:**
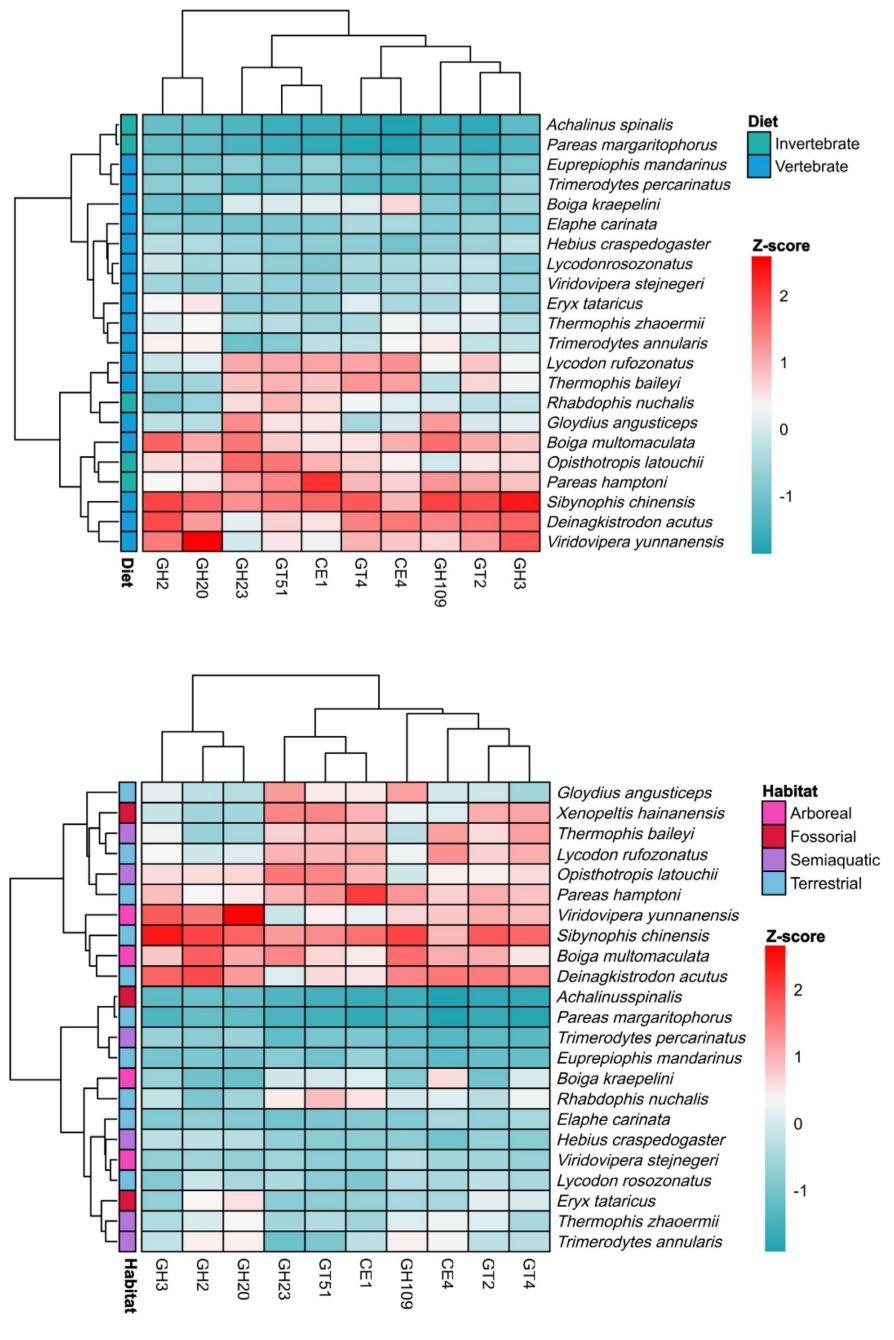
Abundance of the top 10 CAZymes in 23 wild snake species across diet (up) and habitat (down) groups. The abundance value mapping method is *z*‐score.

**FIGURE 9:**
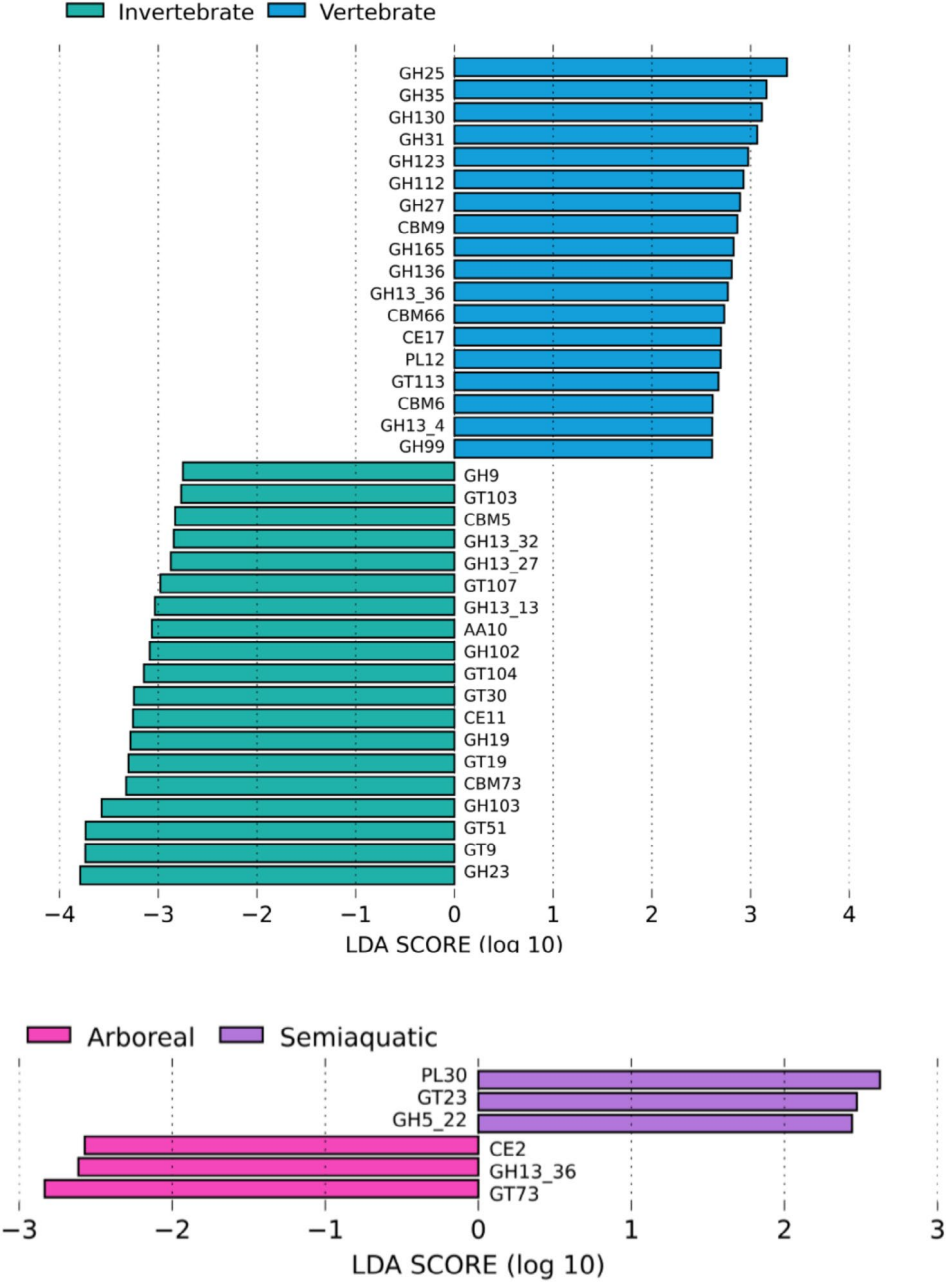
LEfSe analysis of differentially abundant CAZymes in gut microbiota of 23 wild snake species across dietary (up) and habitat (down) groups. LDA scores indicate CAZyme families with significantly different relative abundances between dietary (invertebrate vs. vertebrate, up) and habitat (arboreal vs. fossorial vs. semiaquatic vs. terrestrial, down) groups. Bar length indicates the degree of influence of a CAZyme. Only CAZymes with a significant LDA threshold ≥ 2 are shown.

The four most abundant CAZyme families in the gut microbiota of wild snakes were GT2 (10.19% ± 1.76%), GT4 (6.84% ± 1.69%), GT51 (3.57% ± 0.90%), and GH23 (3.49% ± 1.07%), each with a relative abundance ≥ 3% (Figure 7). This composition remained consistent across both dietary and habitat groups (Figure 8). However, LEfSe analysis revealed distinct CAZyme profiles between groups. Invertebrate‐feeding snakes exhibited 19 unique differential CAZyme families (GH: GT: CBM: AA: CE = 8:7:2:1:1), while vertebrate‐feeding snakes showed 18 (GH: GT: CBM: CE: PL = 12:1:3:1:1) (Figure 9). Among habitat groups, arboreal and semiaquatic species each displayed three unique differential taxa (CE2, GH13_36, GT73 vs. PL30, GT23, GH5_22) (Figure 9).

**FIGURE 10:**
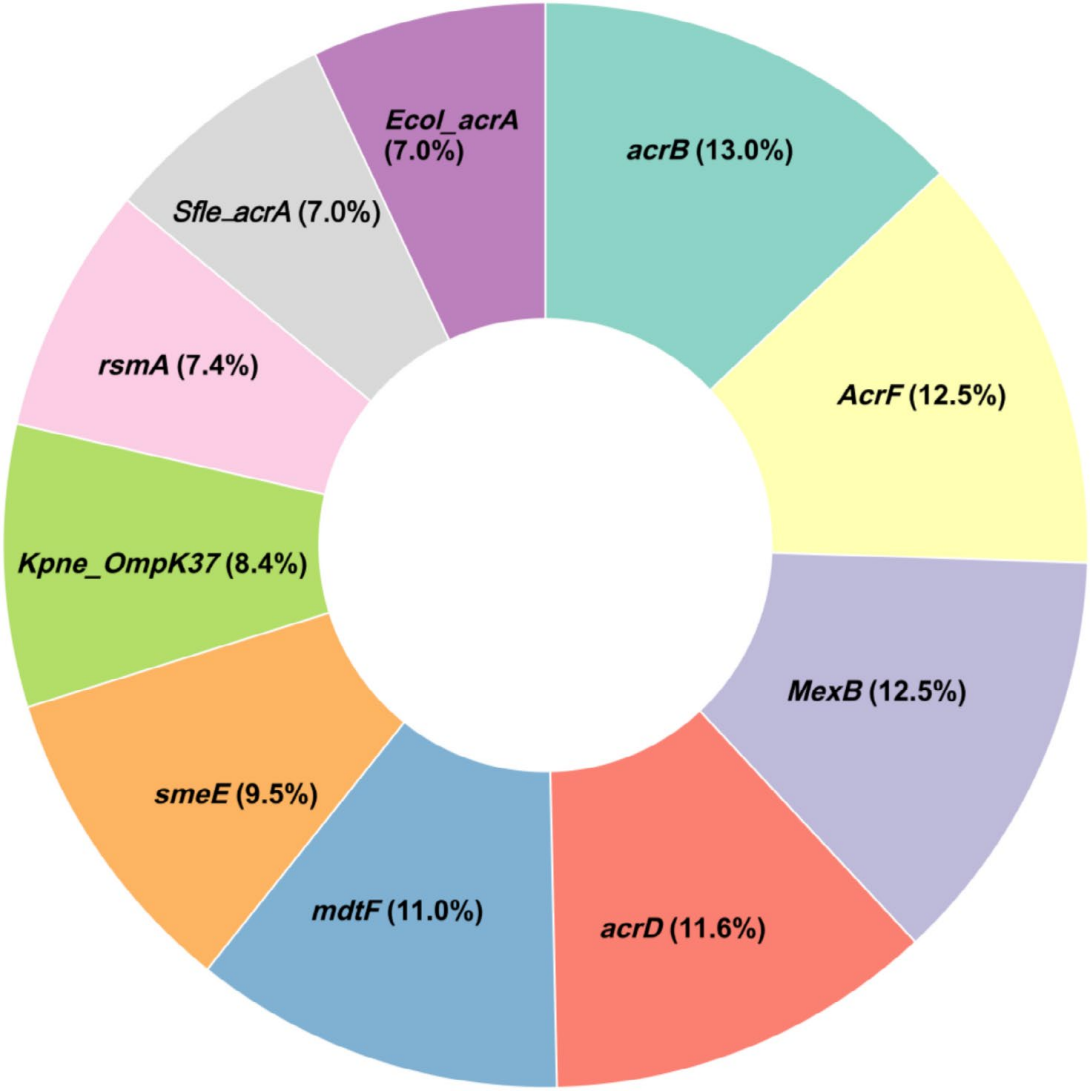
Top 10 most abundant ARGs identified in the gut microbiota of 23 wild snake species.

**FIGURE 11:**
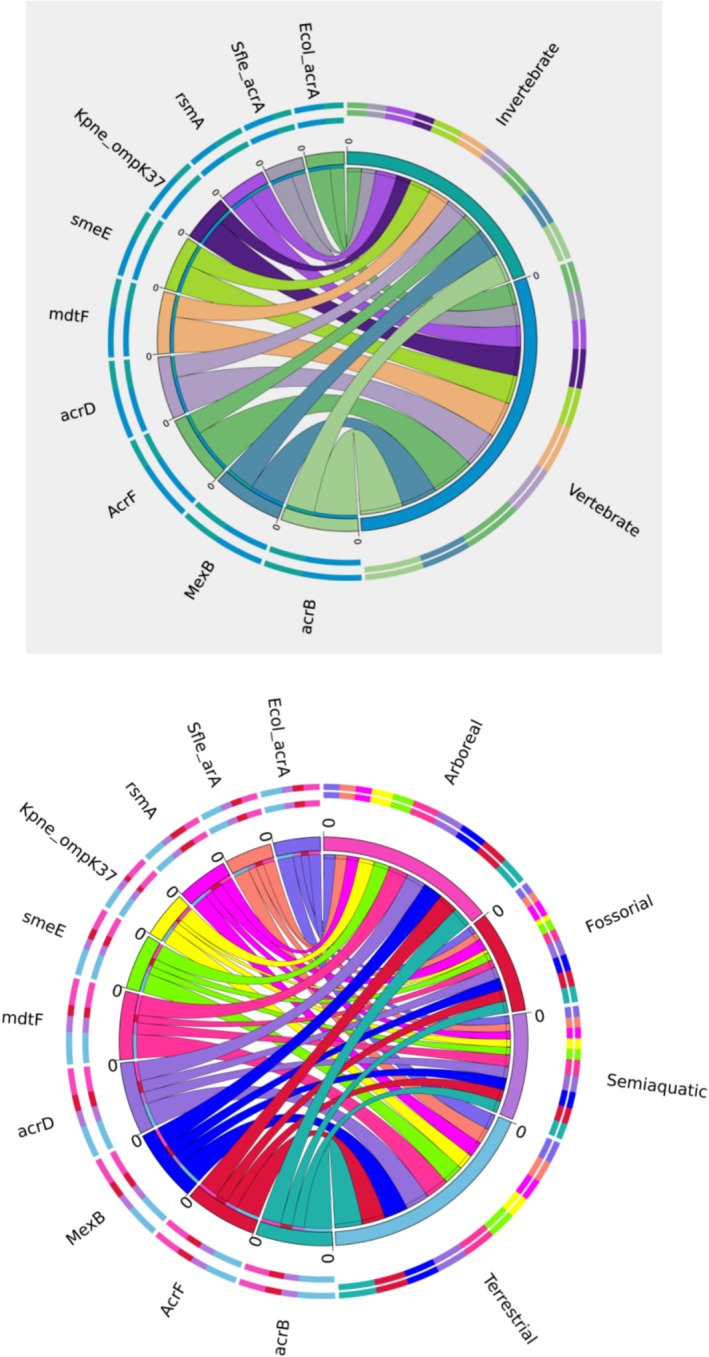
Top 10 most abundant ARGs identified in 23 wild snake species across different dietary (up) and habitat (down) groups. Colors represent different classes. ARGs are shown on the left and different groups on the right. Width of the connecting lines represents the magnitude of abundance.

Analysis of ARGs revealed that the five most abundant were *acrB*, *AcrF*, *MexB*, *acrD*, and *mdtF* (Figure 10). The relative abundance of these resistance genes was higher in vertebrate‐feeding snakes compared to invertebrate‐feeding snakes (Figure 11). Across habitat groups, ARG abundance followed the order: terrestrial > arboreal > fossorial (except for *rsmA*) > semiaquatic (Figure 11).

## 4 Discussion

### 4.1 Gut Microbial Diversity in Wild Snakes

This study represents the first metagenomic next‐generation sequencing to explore diversity of gut microbes in snakes and clarified how phylogeny, diet and habitat affect the gut microbiota based on 23 species of wild snakes from China. Results revealed that Proteobacteria, Bacteroidetes, Firmicutes, and Actinobacteria were the dominant bacterial phyla in the gut microbiota of these snakes (relative abundance ≥ 3%). While previous studies on snakes (wild or captive) showed the gut microbiota are basically Proteobacteria, Bacteroidetes and Firmicutes, with some species having Actinobacteria or Fusobacteria as core flora (Zhang et al. 2024; Zhu et al. 2024). This may reflect its unique biochemical adaptation in the gut of different hosts (Tang, Wang, et al. 2019; Tang, Yang, et al. 2019; Tang, Zhu, et al. 2019). Studies showed that the gut microbiota of lizards is dominated by Bacteroidetes, Firmicutes and Proteobacteria, of which Bacteroidetes and Firmicutes play key roles in vertebrate hosts, with a significant impact on host physiological function at the metabolic and immune levels (Wei et al. 2023; Gao et al. 2024). Proteobacteria has a high and highly variable percentage in the gut microbiota of different lizard species (Zhong et al. 2022; Zhu et al. 2022; Baldo et al. 2023), and a similar situation is found in this study. It arises from the combined effects of the inherent traits of Proteobacteria, including facultative anaerobiosis, broad metabolic versatility and fast growth rate (Chen et al. 2022), together with biological characteristics of lizards such as poikilotherm, simplified intestinal structure, frequent physiological stress, pronounced interspecific variations in diet and habitat, and weak host‐microbe co‐evolutionary relationships (Chen et al. 2022; Gao et al. 2023; Smith et al. 2025).

Species‐specificity in gut microbiota composition is a well‐documented phenomenon across vertebrate clades (Youngblut et al. 2019), with significant interspecific variation observed at the genus level. The dominant genera identified in the present study—*Bacteroides*, *Salmonella*, *Citrobacter*, and *Aeromonas*—are known to include pathogenic strains with potential implications for host physiology and disease susceptibility. *Bacteroides* exhibits dual roles in the host gut environment. Long‐term application of broad‐spectrum antibiotics, hormones, and immunosuppressants to *Bacteroides* can thus lead to endogenous infections due to dysfunctional immunity or dysbiosis of the organisms (Liu 2010; Jiang et al. 2013). On the other hand, *Bacteroides* can also utilize nutrients, regulate nutritional metabolism, and obtain and hydrolyze other indigestible dietary polysaccharides (Costello et al. 2010; Hoffbeck et al. 2025). *Bacteroides fragilis* , the second most abundant *Bacteroides* species detected (10.8% relative abundance), exemplifies this functional dichotomy. As a prominent gut commensal, it contributes to the degradation of complex dietary and host‐derived polysaccharides, supporting host energy metabolism. However, enterotoxigenic strains of *B. fragilis* (ETBF) are also known to produce enterotoxins that disrupt epithelial integrity and induce chronic intestinal inflammation, including colitis (Valguarnera and Wardenburg 2020; Sofi et al. 2021; Eribo et al. 2022; He et al. 2023). Additionally, *Salmonella* and *Citrobacter*, both consistently detected in the gut microbiota of wild snakes (Tang, Wang, et al. 2019, Tang, Yang, et al. 2019, Tang, Zhu, et al. 2019; Zhang et al. 2024), represent key zoonotic taxa. *Salmonella* strains, frequently transmitted between reptiles and humans, are major causative agents of salmonellosis (Grupka et al. 2006; McLaughlin et al. 2015). In this study, *Salmonella enterica* , a primary etiological agent of acute gastroenteritis (Huang et al. 2024), is the most abundant species, accounting for 14.4% of all bacterial sequences. *Aeromonas*, another prevalent genus, includes opportunistic pathogens implicated in a range of infections, such as enteritis and septicemia in both humans and animals (Fernández‐Bravo and Figueras 2020).

### 4.2 Factors Influencing Gut Microbial Diversity

Gut microbial composition in vertebrates is shaped by a complex interplay of factors, including host phylogeny, diet niche, ecological context, geographic location, and age (Wang 2019; Xie et al. 2024; Zhang et al. 2024). Spatial variation within the gastrointestinal tract further contributes to microbial heterogeneity (Waite and Taylor 2014; Tang, Wang, et al. 2019, Tang, Yang, et al. 2019, Tang, Zhu, et al. 2019). Studies have demonstrated that ectotherms are more vulnerable to environmental perturbations than endotherms (Ley et al. 2008; Sepulveda and Moeller 2020). They exhibit pronounced fluctuations in intestinal temperature, which drive seasonal restructuring of gut microbial communities. Furthermore, temperature can modulate the gut microbiota indirectly by regulating host metabolic processes (Tang, Wang, et al. 2019, Tang, Yang, et al. 2019, Tang, Zhu, et al. 2019; Park and Do 2024). A reoccurring pattern in vertebrate systems is the phenomenon of phylosymbiosis, wherein gut microbial assemblages mirror hosts evolutionary relationships, likely mediated by convergent phenotypic traits, digestive morphologies, and ecological constraints (Perofsky et al. 2019; Schmidt et al. 2019; Scheelings et al. 2020). For example, a comparative analysis of seven rodent species revealed striking congruence between host phylogeny and gut microbiota structure, suggesting both phylogenetic and environmental determinants (Zhang et al. 2023). Similarly, an extensive survey across laboratory, urban, and rural mouse populations identified *Kazachstania pintolopesii* as a native fungal symbiont influencing mucosal immunity and systemic physiology (Liao et al. 2024). Nevertheless, gut microbial communities of lizards exhibit stronger seasonal divergence than interspecific variation, indicating relatively weak “phylogenetic symbiosis” pattern in ectothermic vertebrates (Bestion et al. 2017; Sharpton 2018; Yang et al. 2024). This study indicated that at the phylum level, there were no significant differences in the impact of phylogeny on gut microbial diversity in snakes, and the composition of gut microbes has some similarity. While, there was a significant correlation between genetic distance (*p*‐distance) and gut microbiota at the general level, which might be consistent with the pattern of “phylogenetic symbiosis”. But the Pcoa analysis showed that the overall level of gut microbiota was not affected by the host phylogeny. This is influenced by the sample size.

The PCoA results further indicated that dietary partitioning and habitat exerted no effect different taxonomic level (e.g., suborder). But Comparable patterns have been reported in bovines, where microbial community profiles are more strongly associated with diet than host taxonomy at fine phylogenetic resolutions (Rojas et al. 2021). More studies have also showed that the gut microbiota of proximate hosts is more influenced by host ecology than by host phylogeny. A study of 14 baboon populations found that differences in gut microbes did not increase with host genetic distance, but did increase with habitat soil effects (Grieneisen et al. 2019). Studies on reptiles have demonstrated that diet and habitat, instead of host phylogeny, serve as the predominant drivers shaping gut microbial community profiles (Smith et al. 2025; Zhu et al. 2025). While LEfSe analysis revealed significant differences in microbial abundance across dietary and habitat groups. These results may be related to the uneven distribution of the sample size collected in this study. In the future, the samples should be increased to analyze the relationship between gut microbes and hosts from different taxonomic groups, which will help to gain more in‐depth information about gut microbes in wild snakes.

### 4.3 Functions of Gut Microbes in Wild Snakes

These functions of the gut microbes of 23 wild snake species were primarily associated with metabolic activity and the processing of environmental and genetic information, mainly including membrane transport, energy metabolism, signal transduction, nucleotide metabolism, etc. This is basically consistent with previous research on snakes (Li et al. 2021; Tang, Wang, et al. 2019; Tang, Yang, et al. 2019; Tang, Zhu, et al. 2019).

Carbohydrate metabolism constitutes a major functional axis of gut microbial activity in snakes (McLaughlin et al. 2015; Tang, Wang, et al. 2019; Tang, Yang, et al. 2019; Tang, Zhu, et al. 2019; Li et al. 2021), yet the enzymatic machinery underpinning this process, particularly CAZymes, remains poorly characterized. In the present study, GT2, GT4, GT51, and GH23 emerged as the most abundant CAZyme families within the gut microbiota of 23 wild snake species. The three most abundant enzymes (GT2, GT4, and GT51) belong to the glycosyl transferase (GT) superfamily, which mediates the biosynthesis of polysaccharides, glycoproteins, and other glycoconjugates, playing essential roles in cell wall assembly and molecular signaling (Zhu et al. 2015). GH23 primarily mediates peptidoglycan degradation during bacterial cell wall remodeling and bacteriophage invasion and also includes immune‐related molecules potentially involved in immunomodulation (Hobbs et al. 2018). LEfSe analysis revealed distinct CAZyme profiles across dietary and habitat groups, with diet exerting a stronger influence than habitat. Notably, vertebrate‐feeding snakes exhibited a pronounced enrichment of glycoside hydrolases (GH) family members (*n* = 12), mirroring functional trends observed in mammalian systems where GHs drive polysaccharide degradation and nutrient assimilation, as observed in bovine rumen studies (Liu et al. 2023). These findings suggest that GH enzymes similarly support digestive adaptation in vertebrate‐eating snakes, a hypothesis warranting further investigation through integrated metabolomic and transcriptomic analyses to elucidate expression dynamics and regulatory control. In humans, GH and polysaccharide lyase (PL) enzymes involved in glycosidic bond cleavage are predominantly encoded by Bacteroidetes and Firmicutes, underscoring their central role in host carbohydrate metabolism (Onyango et al. 2021). Given the observed host‐microbe phylogenetic correlation in this study, it is plausible that variation in snake CAZyme repertoires reflects evolutionary divergence in host digestive strategies. However, this experiment did not further analyze the differences in gene functions among different groups, and it is necessary to conduct a more in‐depth analysis of the substrates on which the advantageous CAZymes act in order to better interpret it from the perspective of diet.

The composition and structural dynamics of the gut microbiota serve as key indicators of host health and play critical roles in disease diagnosis, prevention, and treatment outcomes (Kundu et al. 2017; Rosshart et al. 2017; Hu et al. 2017). ARGs harbored by gut microbes provide important insights into host exposure to antibiotics and the potential for zoonotic transmission. Multiple studies have documented the presence of conditionally pathogenic bacteria in the intestinal microflora of various animals (Tang, Wang, et al. 2019; Tang, Yang, et al. 2019; Tang, Zhu, et al. 2019; Liu et al. 2024; Zhang et al. 2024). And ectotherms carrying pathogenic bacteria and viruses tend to facilitate environmental transmission of such pathogens, which consequently poses potential threats to human health (Loh et al. 2015). This rise in antibiotic resistance often leads to treatment failure, promotes pathogen dissemination, and disrupts gut microbial homeostasis. In this study, the five most abundant ARGs were *acrB*, *AcrF*, *MexB*, *acrD*, and *mdtF*, which are associated with bacterial resistance to tetracyclines, β‐lactams, and quinolones. And antibiotic‐resistant taxa identified included *Salmonella enterica* , *Bacteroides fragilis* , *Citrobacter freundii* , and *Pseudomonas aeruginosa* . Notably, *MexB* and *acrD* are known to confer chloramphenicol resistance in *Pseudomonas aeruginosa* (Sirjana et al. 2022), a bacterium widely distributed in soil environments.

Comparative analyses revealed that ARG abundance was consistently higher in vertebrate‐feeding snakes compared to invertebrate‐feeding snakes. Across habitat types, ARG levels followed the pattern: terrestrial > arboreal > burrowing (except for *rsmA*) > semiaquatic. These findings suggest that both dietary strategy and habitat niche modulate ARG composition. Supporting evidence from murine studies has shown that high‐fat diets can compromise antibiotic efficacy by reshaping gut flora composition and metabolite profiles (Li et al. 2021). Furthermore, environmental microorganisms (in water or soil) are important reservoirs for zoonotic diseases related to ectotherms (Abia Akebe et al. 2022; Leifels et al. 2022). Therefore, monitoring ARGs in the gut microbiota of wild snakes holds relevance for both conservation biology and biosecurity management.

## 5 Conclusions

Metagenomic analysis of the gut microbiota of 23 wild snake species identified a total of 20,341 bacterial taxa (species) spanning 49 phyla, 82 classes, 187 orders, 439 families, and 2428 genera. The dominant phyla were Proteobacteria, Bacteroidetes, Firmicutes, and Actinobacteria, although certain species exhibited core enrichment of Actinobacteria or Fusobacteria, likely shaped by local environmental conditions. The dominant genera were *Bacteroides*, *Salmonella*, *Citrobacter*, and *Aeromonas*. A significant correlation between host phylogeny (*p*‐distance) and gut microbial composition was observed at the genus level, consistent with the pattern of phylogenetic symbiosis. Functional annotation indicated enrichment of genes involved in metabolism, environment sensing, and genetic information processing, with functional variation likely linked to dietary classification. The most prevalent CAZyme families, GT2, GT4, GT51, and GH23, were conserved across dietary and habitat groups, although differential abundances of specific enzymes varied between groups. The five most abundant ARGs were *acrB*, *AcrF*, *MexB*, *acrD*, and *mdtF*, with relative abundances influenced by both diet and habitat. Analyses of α and β diversity revealed phylogeny, diet, and habitat did not significantly differentiate the gut microbes of snakes at different taxonomic scales. Future studies should increase the sample numbers and comprehensively consider different ecological factors to explore the impacts on the composition and functions of snake gut microbes across various taxonomic groups.

## Author Contributions

**Jiaqi Zhang:** conceptualization (lead), formal analysis (equal), methodology (equal), visualization (lead), writing – original draft (lead). **Peng Guo:** conceptualization (lead), funding acquisition (equal), methodology (equal), resources (equal), supervision (equal), visualization (equal), writing – review and editing (equal). **Bing Lyu:** data curation (equal), writing – review and editing (supporting). **Lei Shi:** conceptualization (equal), methodology (equal), supervision (equal), visualization (equal), writing – review and editing (equal). **Songwen Tan:** formal analysis (equal), supervision (equal), visualization (equal). **Yayong Wu:** conceptualization (equal), data curation (equal), formal analysis (equal), supervision (lead), writing – review and editing (lead). **Guocheng Shu:** conceptualization (equal), data curation (equal), writing – review and editing (equal). **Chunmei Fu:** formal analysis (equal), methodology (equal), visualization (equal).

## Funding

This work was funded by the Third Xinjiang Comprehensive Scientific Expedition, the Ministry of Science and Technology of PRC (2022xjkk0801), National Natural Science Foundation of China (NSFC 32370486), the Second Tibetan Plateau Scientific Expedition and Research (STEP) Program (2024QZKK0200, 2019QZKK05010105), and Comprehensive Scientific Survey of Huanglong Nature Reserve, Huanglong National Scenic Area Administration, Sichuan, China (N5132112023000495).

## Ethics Statement

Sample collection was approved by the Animal Care and Ethics Committee of Yibin University (YBU2020007).

## Consent

The authors have nothing to report.

## Conflicts of Interest

The authors declare no conflicts of interest.

## Supporting information


**Appendix S1:** Data collected from 73 individuals.

**Appendix S2:** Downloading sequences from NCBI.

**Appendix S3:** Phylogenetic relationship.

**Appendix S4:** Description of the feeding habits of 23 species of wild snakes.

## Data Availability

The data supporting the findings of this study are publicly available from the NCBI Sequence Read Archive (SRA) at https://www.ncbi.nlm.nih.gov/bioproject/PRJNA1108923, https://www.ncbi.nlm.nih.gov/bioproject/PRJNA1108898 (SRA accession: PRJNA1108898–PRJNA1108923), https://dataview.ncbi.nlm.nih.gov/object/PRJNA1144187 (SRA accession: SRR30144259–SRR30144282 and SRR30144402–SRR30144425), https://www.ncbi.nlm.nih.gov/bioproject/PRJNA1108955 (SRA accession: SRR29213544–SRR29213564), and https://www.ncbi.nlm.nih.gov/sra/SRR29924189.
