# Supplementary material for: How Host Phylogeny, Diet, and Habitat Affect Gut Microbial Diversity in Wild Snakes
Source: Ecol Evol. 2026 Jul 1;16(7):e73902. doi: 10.1002/ece3.73902 (PMC13322667; doi:10.1002/ece3.73902)
Supplement: Supplementary file 1 — Appendix S1: Data collected from 73 individuals. [file ECE3-16-e73902-s001.docx]

**Appendix SI Data collected from 73 individuals**

| **Family** | **Genus** | **Species** | **Sample ID** | **Sex** | **Collection sites** | **Longitude** | **Latitude** | **Altitude** |
| --- | --- | --- | --- | --- | --- | --- | --- | --- |
| Boidae | *Eryx* | *Eryx tataricus* | SRR30144403 | / | Jinghe, Bortala, Xinjiang | 83°18″ | 44°40′ | 301 |
| Colubridae | *Boiga* | *Boiga kraepelini* | SRR30144263 | ♂ | Huangshan District, City, Anhui | 118°02′ | 30°13′ | 258 |
| Colubridae | *Boiga* | *Boiga multomaculata* | SRR30144266 | ♂ | Xichou, Wenshan, Yunnan | 104°49′ | 23°39′ | / |
| Colubridae | *Elaphe* | *Elaphe carinata* | SRR30144270 | ♀ | Huangshan District, City, Anhui | 118°01′ | 30°14′ | 248 |
| Colubridae | *Elaphe* | *Elaphe carinata* | SRR30144279 | ♀ | Huangshan District, City, Anhui | 118°08′ | 30°18′ | 270 |
| Colubridae | *Elaphe* | *Elaphe carinata* | SRR30144424 | ♂ | Batang, Garze, Sichuan | 99°04″ | 29°01′ | / |
| Colubridae | *Euprepiophis* | *Euprepiophis mandarinus* | SRR30144267 | / | Xifeng, Guiyang, Guizhou | 106°28′ | 21°06′ | 1079 |
| Colubridae | *Euprepiophis* | *Euprepiophis mandarinus* | SRR30144405 | ♂ | Xuyong, Luzhou, Sichuan | 105°56′ | 28°23′ | 783 |
| Colubridae | *Euprepiophis* | *Euprepiophis mandarinus* | SRR30144408 | ♂ | Xuyong, Luzhou, Sichuan | 105°56′ | 28°23′ | 783 |
| Colubridae | *Lycodon* | *Lycodon rufozonatus* | SRS21455565 | ♀ | Xifeng, Guiyang, Guizhou | 106°49′ | 27°38′ | 970 |
| Colubridae | *Lycodon* | *Lycodon rufozonatus* | SRS21455566 | ♀ | Xifeng, Guiyang, Guizhou | 106°49′ | 27°38′ | 968 |
| Colubridae | *Lycodon* | *Lycodon rufozonatus* | SRS21455563 | / | Xifeng, Guiyang, Guizhou | 106°49′ | 27°11′ | 970 |
| Colubridae | *Lycodon* | *Lycodon rufozonatus* | SRS21455560 | / | Guiyang, Guizhou | 106°55′ | 26°52′ | / |
| Colubridae | *Lycodon* | *Lycodon rufozonatus* | SRS21455561 | / | Guiyang, Guizhou | 106°55′ | 26°52′ | / |
| Colubridae | *Lycodon* | *Lycodon rosozonatus* | SRS21455546 | / | Lingshui, Hainan | 109°52′ | 18°53′ | / |
| Colubridae | *Lycodon* | *Lycodon rosozonatus* | SRS21455547 | / | Lingshui, Hainan | 109°52′ | 18°53′ | / |
| Colubridae | *Lycodon* | *Lycodon rosozonatus* | SRS21455557 | / | Lingshui, Hainan | 109°53′ | 18°53′ | / |
| Colubridae | *Lycodon* | *Lycodon rufozonatus* | SRS21455548 | / | Yongxing, Chenzhou, Hunan | 113°21′ | 26°15′ | 250 |
| Colubridae | *Lycodon* | *Lycodon rufozonatus* | SRS21455549 | / | Yongxing, Chenzhou, Hunan | 113°00′ | 26°05′ | 155 |
| Colubridae | *Lycodon* | *Lycodon rufozonatus* | SRS21455551 | / | Yongxing, Chenzhou, Hunan | 113°58′ | 26°04′ | 128 |
| Colubridae | *Lycodon* | *Lycodon rufozonatus* | SRS21455550 | ♀ | Yongxing, Chenzhou, Hunan | 113°06′ | 26°07′ | / |
| Colubridae | *Lycodon* | *Lycodon rufozonatus* | SRS21455554 | ♂ | Yongxing, Chenzhou, Hunan | 113°06′ | 26°07′ | / |
| Dipsadidae | *Thermophis* | *Thermophis zhaoermii* | SRR30144415 | / | Litang, Garze, Sichuan | 100°08″ | 30°03′ | 4039 |
| Dipsadidae | *Thermophis* | *Thermophis zhaoermii* | SRR30144416 | ♂ | Litang, Garze, Sichuan | 100°08″ | 30°03′ | 4039 |
| Dipsadidae | *Thermophis* | *Thermophis baileyi* | SRR30144417 | ♂ | Litang, Garze, Sichuan | 99°51″ | 30°24′ | / |
| Dipsadidae | *Thermophis* | *Thermophis baileyi* | SRR30144418 | ♀ | Litang, Garze, Sichuan | 99°50″ | 30°24′ | / |
| Natricidea | *Hebius* | *Hebius craspedogaster* | SRR30144268 | ♀ | Hejiang, Luzhou, Sichuan | 106°14″ | 28°04′ | / |
| Natricidea | *Hebius* | *Hebius craspedogaster* | SRR30144281 | ♀ | Xuyong, Luzhou, Sichuan | 105°56′ | 28°23′ | 783 |
| Natricidea | *Hebius* | *Hebius craspedogaster* | SRR30144282 | / | Xuyong, Luzhou, Sichuan | 105°56′ | 28°23′ | 783 |
| Natricidea | *Hebius* | *Hebius craspedogaster* | SRR30144407 | ♀ | Xuyong, Luzhou, Sichuan | 105°56′ | 28°23′ | 783 |
| Natricidea | *Opisthotropis* | *Opisthotropis latouchii* | SRR30144419 | ♀ | Huangshan District, City, Anhui | 118°01′ | 30°16′ | 191 |
| Natricidea | *Opisthotropis* | *Opisthotropis latouchii* | SRR30144420 | ♀ | Huangshan District, City, Anhui | 118°01′ | 30°16′ | 191 |
| Natricidea | *Opisthotropis* | *Opisthotropis latouchii* | SRR30144421 | ♂ | Huangshan District, City, Anhui | 118°01′ | 30°14′ | 190 |
| Natricidea | *Rhabdophis* | *Rhabdophis nuchalis* | SRR30144261 | ♀ | Heishui, Aba, Sichuan | 102°52″ | 32°02′ | 2500 |
| Natricidea | *Rhabdophis* | *Rhabdophis nuchalis* | SRR30144262 | ♀ | Heishui, Aba, Sichuan | 102°52″ | 32°02′ | 2500 |
| Natricidea | *Rhabdophis* | *Rhabdophis nuchalis* | SRR30144271 | ♀ | Heishui, Aba, Sichuan | 102°52″ | 32°02′ | / |
| Natricidea | *Trimerodytes* | *Trimerodytes annularis* | SRR30144423 | ♀ | Huangshan District, City, Anhui | 118°08′ | 30°18′ | 195 |
| Natricidea | *Trimerodytes* | *Trimerodytes annularis* | SRR30144411 | / | Yongxing, Chenzhou, Hunan | 113°06′ | 26°07′ | / |
| Natricidea | *Trimerodytes* | *Trimerodytes percarinatus* | SRR30144276 | ♀ | Huangshan District, City, Anhui | 118°01′ | 30°14′ | 174 |
| Natricidea | *Trimerodytes* | *Trimerodytes percarinatus* | SRR30144422 | ♂ | Huangshan District, City, Anhui | 118°01′ | 30°15′ | 204 |
| Natricidea | *Trimerodytes* | *Trimerodytes percarinatus* | SRR30144406 | / | Xuyong, Luzhou, Sichuan | 105°56′ | 28°23′ | 783 |
| Natricidea | *Trimerodytes* | *Trimerodytes percarinatus* | SRR30144413 | / | Xuyong, Luzhou, Sichuan | 105°56′ | 28°23′ | 783 |
| Pareidae | *Pareas* | *Pareas hamptoni* | SRR30144274 | ♂ | Pingbian, Honghe, Yunnan | 103°42′ | 22°57′ | 1616 |
| Pareidae | *Pareas* | *Pareas hamptoni* | SRR30144275 | ♀ | Pingbian, Honghe, Yunnan | 103°42′ | 22°57′ | 1616 |
| Pareidae | *Pareas* | *Pareas margaritophorus* | SRR30144273 | ♀ | Xichou, Wenshan, Yunnan | 104°47′ | 23°22′ | 1359 |
| Sibynophiidae | *Sibynophis* | *Sibynophis chinensis* | SRR30144410 | / | Ningyuan, Yongzhou, Hunan | 111°57′ | 25°34′ | / |
| Sibynophiidae | *Sibynophis* | *Sibynophis chinensis* | SRR30144414 | ♀ | Naxi, Lijiang, Yunnan | 100°04″ | 27°21′ | / |
| Viperidae | *Deinagkistrodon* | *Deinagkistrodon acutus* | SRR30144277 | / | Huangshan District, City, Anhui | 118°00′ | 30°16′ | 230 |
| Viperidae | *Deinagkistrodon* | *Deinagkistrodon acutus* | SRR30144278 | ♀ | Huangshan District, City, Anhui | 118°01′ | 30°16′ | 181 |
| Viperidae | *Deinagkistrodon* | *Deinagkistrodon acutus* | SRR30144402 | ♀ | Huangshan District, City, Anhui | 118°01′ | 31°18′ | / |
| Viperidae | *Deinagkistrodon* | *Deinagkistrodon acutus* | SRR30144409 | / | Huangshan District, City, Anhui | 118°19′ | 29°43′ | / |
| Viperidae | *Gloydius* | *Gloydius angusticeps* | SRR30144259 | / | Rangtang, Aba, Sichuan | 102°56″ | 32°26′ | 3586 |
| Viperidae | *Gloydius* | *Gloydius angusticeps* | SRR30144260 | / | Rangtang, Aba, Sichuan | 102°56″ | 32°26′ | 3586 |
| Viperidae | *Gloydius* | *Gloydius angusticeps* | SRR30144280 | / | Rangtang, Aba, Sichuan | 102°56″ | 32°26′ | 3586 |
| Viperidae | *Viridovipera* | *Viridovipera stejnegeri* | SRR28961925 | ♂ | Huangshan District, City, Anhui | 118°00′ | 30°14′ | 216 |
| Viperidae | *Viridovipera* | *Viridovipera stejnegeri* | SRR28961926 | ♀ | Huangshan District, City, Anhui | 118°02′ | 30°14′ | 209 |
| Viperidae | *Viridovipera* | *Viridovipera stejnegeri* | SRR28961927 | ♂ | Huangshan District, City, Anhui | 118°00′ | 30°14′ | 197 |
| Viperidae | *Viridovipera* | *Viridovipera stejnegeri* | SRR28961928 | ♀ | Huangshan District, City, Anhui | 118°01′ | 30°14′ | 220 |
| Viperidae | *Viridovipera* | *Viridovipera stejnegeri* | SRR28961929 | ♀ | Huangshan District, City, Anhui | 118°01′ | 30°14′ | 220 |
| Viperidae | *Viridovipera* | *Viridovipera stejnegeri* | SRR28961930 | ♂ | Huangshan District, City, Anhui | 118°01′ | 30°14′ | 200 |
| Viperidae | *Viridovipera* | *Viridovipera stejnegeri* | SRR28961923 | ♂ | Kaiyang, Guiyang, Guizhou | 106°49′ | 27°11′ | 942 |
| Viperidae | *Viridovipera* | *Viridovipera stejnegeri* | SRR28961924 | ♂ | Kaiyang, Guiyang, Guizhou | 106°47′ | 27°05′ | 1203 |
| Viperidae | *Viridovipera* | *Viridovipera stejnegeri* | SRR28961931 | ♀ | Guiyang, Guizhou | 106°54′ | 26°28′ | 1232 |
| Viperidae | *Viridovipera* | *Viridovipera stejnegeri* | SRR28961932 | ♂ | Guiyang, Guizhou | 106°54′ | 26°28′ | 1232 |
| Viperidae | *Viridovipera* | *Viridovipera stejnegeri* | SRR28961933 | ♀ | Guiyang, Guizhou | 106°54′ | 26°28′ | 1232 |
| Viperidae | *Viridovipera* | *Viridovipera stejnegeri* | SRR28961175 | ♂ | Yongxing, Chenzhou, Hunan | 113°23′ | 26°14′ | 318 |
| Viperidae | *Viridovipera* | *Viridovipera stejnegeri* | SRR28961934 | ♂ | Yongxing, Chenzhou, Hunan | 113°22′ | 26°15′ | 319 |
| Viperidae | *Viridovipera* | *Viridovipera stejnegeri* | SRR28961935 | ♀ | Yongxing, Chenzhou, Hunan | 113°23′ | 26°14′ | 318 |
| Viperidae | *Viridovipera* | *Viridovipera yunnanensis* | SRR30144425 | ♀ | Huili, Liangshan, Sichuan | 102°04″ | 27°14′ | / |
| Xenodermidae | *Achalinus* | *Achalinus spinalis* | SRR30144412 | / | Emeishan, Sichuan | 103°21″ | 29°14′ | / |
| Xenodermidae | *Achalinus* | *Achalinus spinalis* | SRR30144404 | ♂ | Yucheng District, Ya 'an, Sichuan | 103°01′ | 30°00′ | 783 |
| Xenopeltidae | *Xenopeltis* | *Xenopeltis hainanensis* | SRR30144264 | ♂ | Nanning, Guangxi | 108°23′ | 22°08′ | / |
| Xenopeltidae | *Xenopeltis* | *Xenopeltis hainanensis* | SRR30144265 | ♂ | Hainan | 110°14′ | 20°15′ | / |
