# Supplementary material for: How Host Phylogeny, Diet, and Habitat Affect Gut Microbial Diversity in Wild Snakes
Source: Ecol Evol. 2026 Jul 1;16(7):e73902. doi: 10.1002/ece3.73902 (PMC13322667; doi:10.1002/ece3.73902)
Supplement: Supplementary file 2 — Appendix S2: Downloading sequences from NCBI. [file ECE3-16-e73902-s004.docx]

**Appendix S2 Downloading sequences from NCBI**

| **Species** | **Host reference genome** | **CYTB** | **COI** | **ND2** | **ND4** |
| --- | --- | --- | --- | --- | --- |
| *Achalinus spinalis* | *Acrochordus granulatus* | MK201476.1 | MK064822.1 | MK199033.1 | NC_032084.1 |
| *Boiga kraepelini* | *Ptyas mucosa* | MN962360.1 | MG788988.1 | MK199001.1 | NC_070009.1 |
| *Boiga multomaculata* | *Ptyas mucosa* | ON932788.1 | MK064860.1 | MN962404.1 | ON932765.1 |
| *Deinagkistrodon acutus* | *Protobothrops mucrosquamatus* | KX694847.1 | KR046071.1 | DQ836196.1 | AY352811.1 |
| *Elaphe carinata* | *Pantherophis guttatus* | LC327647.1 | JF700159.1 | JN799418.1 | JN799414.1 |
| *Eryx tataricus* | *Charina bottae* | KF811117.1 | MK064714.1 | MK198947.1 | KF576680.1 |
| *Euprepiophis mandarinus* | *Pantherophis guttatus* | DQ902115.1 | KF698942.1 | DQ902222.1 | DQ902294.1 |
| *Gloydius angusticeps* | *Protobothrops mucrosquamatus* | SRR29924189 | OQ416168.1 | / | / |
| *Hebius craspedogaster* | *Natrix natrix* | KJ685704.1 | MK064769.1 | MK198988.1 | NC_070008.1 |
| *Lycodon rosozonatus* | *Ptyas mucosa* | MK201531.1 | OQ416368.1 | MK199090.1 | KC733229.1 |
| *Lycodon rufozonatus* | *Ptyas mucosa* | AF471063.1 | JQ798868.1 | MK199040.1 | / |
| *Opisthotropis latouchii* | *Natrix natrix* | GQ281783.1 | MK064905.1 | MK199098.1 | JQ687421.1 |
| *Pareas hamptoni* | *Protobothrops mucrosquamatus* | MW287077.1 | OQ416630.1 | MK198847.1 | PP215396.1 |
| *Pareas margaritophorus* | *Protobothrops mucrosquamatus* | MZ712217.1 | OQ416619.1 | / | MZ712243.1 |
| *Rhabdophis nuchalis* | *Rhabdophis nuchalis* | KF800934.1 | MK064703.1 | MK198937.1 | OQ868261.1 |
| *Sibynophis chinensis* | *Sibynophis collaris* | MW199781.1 | KU529421.1 | KC000131.1 | NC_022430.1 |
| *Thermophis baileyi* | *Thermophis baileyi* | KF595119.1 | MK064712.1 | JF411072.1 | KF595097.1 |
| *Thermophis zhaoermii* | *Thermophis baileyi* | EU864146.1 | MK064710.1 | JF411071.1 | JN564717.1 |
| *Trimerodytes annularis* | *Natrix natrix* | MN017778.1 | KR045998.1 | MN582512.1 | JQ687424.1 |
| *Trimerodytes percarinatus* | *Natrix natrix* | MN017780.1 | MF099683.1 | MN582516.1 | JQ687426.1 |
| *Viridovipera stejnegeri* | *Protobothrops mucrosquamatus* | EF597524.1 | KR045963.1 | MK199049.1 | EF597528.1 |
| *Viridovipera yunnanensis* | *Protobothrops mucrosquamatus* | EF597523.1 | MK064801.1 | MK199012.1 | KX019295.1 |
| *Xenopeltis hainanensis* | *Python bivittatus* | MK201533.1 | MK064884.1 | MK199092.1 | / |
| *Argyrophis diardii* | / | KT316507.1 | OQ416981.1 | / | / |
| *Indotyphlops braminus* | / | KT316548.1 | JQ909572.1 | NC_010196.1:3743-4775 | / |

Note: Host reference sequences were used for de-hosting comparisons in data analysis. COI, CYTB, ND2, and ND4 sequences were used to construct the BI tree for 23 snake species.
