# Supplementary material for: How Host Phylogeny, Diet, and Habitat Affect Gut Microbial Diversity in Wild Snakes
Source: Ecol Evol. 2026 Jul 1;16(7):e73902. doi: 10.1002/ece3.73902 (PMC13322667; doi:10.1002/ece3.73902)
Supplement: Supplementary file 3 — Appendix S3: Phylogenetic relationship. [file ECE3-16-e73902-s002.docx]

**Appendix S3 Phylogenetic relationship**

The phylogenetic relationships among the 23 snake species, reconstructed from the combined database (Figure 1) were consistent with previously published findings (Li *et al*., 2020). Pairwise uncorrected genetic distances (*p*-distance) between species are shown in Table 1.


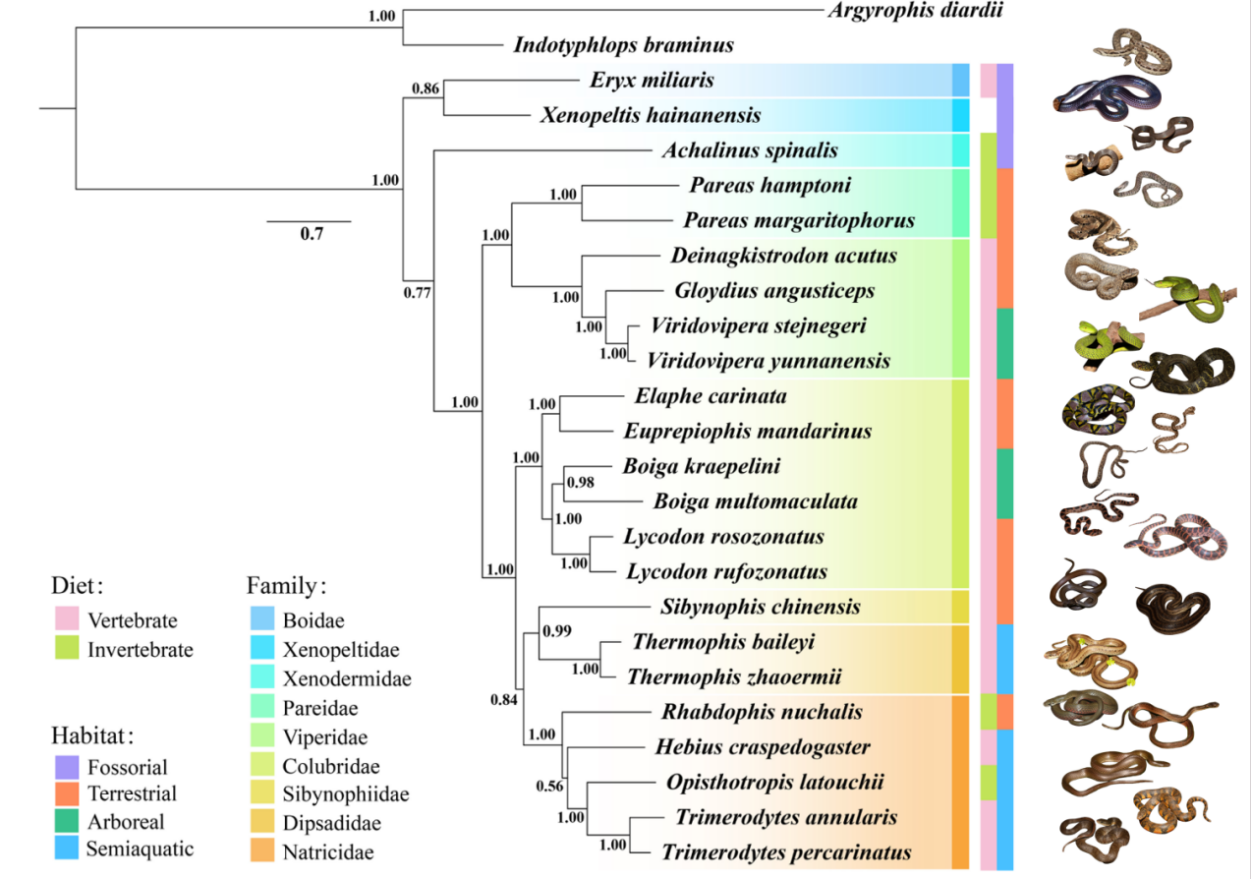


**Figure 1 Bayesian phylogenetic tree of 23 wild snake species constructed from the combined database including COI, CYTB, ND2, and ND4**

**Table 1 Pairwise uncorrected genetic distances (*p*-distance) based on COI**

| Number | Species | 1 | 2 | 3 | 4 | 5 | 6 | 7 | 8 | 9 | 10 | 11 | 12 |
| --- | --- | --- | --- | --- | --- | --- | --- | --- | --- | --- | --- | --- | --- |
| 1 | *Eryx tataricus* |  |  |  |  |  |  |  |  |  |  |  |  |
| 2 | *Xenopeltis hainanensis* | 0.172 |  |  |  |  |  |  |  |  |  |  |  |
| 3 | *Achalinus spinalis* | 0.218 | 0.190 |  |  |  |  |  |  |  |  |  |  |
| 4 | *Pareas hamptoni* | 0.236 | 0.243 | 0.234 |  |  |  |  |  |  |  |  |  |
| 5 | *Pareas margaritophorus* | 0.223 | 0.225 | 0.229 | 0.209 |  |  |  |  |  |  |  |  |
| 6 | *Deinagkistrodon acutus* | 0.195 | 0.205 | 0.209 | 0.230 | 0.200 |  |  |  |  |  |  |  |
| 7 | *Gloydius angusticeps* | 0.192 | 0.196 | 0.205 | 0.203 | 0.211 | 0.158 |  |  |  |  |  |  |
| 8 | *Viridovipera stejnegeri* | 0.192 | 0.196 | 0.195 | 0.223 | 0.203 | 0.142 | 0.136 |  |  |  |  |  |
| 9 | *Viridovipera yunnanensis* | 0.177 | 0.190 | 0.195 | 0.223 | 0.203 | 0.142 | 0.118 | 0.044 |  |  |  |  |
| 10 | *Elaphe carinata* | 0.192 | 0.174 | 0.216 | 0.207 | 0.212 | 0.204 | 0.191 | 0.198 | 0.178 |  |  |  |
| 11 | *Euprepiophis mandarinus* | 0.174 | 0.155 | 0.210 | 0.221 | 0.201 | 0.172 | 0.172 | 0.172 | 0.166 | 0.154 |  |  |
| 12 | *Boiga kraepelini* | 0.177 | 0.187 | 0.221 | 0.218 | 0.212 | 0.201 | 0.203 | 0.190 | 0.192 | 0.143 | 0.134 |  |
| 13 | *Boiga multomaculata* | 0.186 | 0.169 | 0.213 | 0.218 | 0.214 | 0.201 | 0.200 | 0.193 | 0.186 | 0.169 | 0.152 | 0.154 |
| 14 | *Lycodon rosozonatus* | 0.183 | 0.187 | 0.211 | 0.220 | 0.209 | 0.176 | 0.192 | 0.185 | 0.183 | 0.174 | 0.140 | 0.156 |
| 15 | *Lycodon rufozonatus* | 0.195 | 0.187 | 0.195 | 0.214 | 0.209 | 0.199 | 0.200 | 0.193 | 0.186 | 0.157 | 0.154 | 0.166 |
| 16 | *Sibynophis chinensis* | 0.205 | 0.190 | 0.209 | 0.214 | 0.218 | 0.219 | 0.203 | 0.210 | 0.209 | 0.189 | 0.180 | 0.195 |
| 17 | *Termophis baileyi* | 0.202 | 0.190 | 0.204 | 0.201 | 0.212 | 0.192 | 0.200 | 0.183 | 0.172 | 0.175 | 0.183 | 0.174 |
| 18 | *Thermophis zhaoermii* | 0.212 | 0.195 | 0.221 | 0.214 | 0.192 | 0.190 | 0.189 | 0.202 | 0.198 | 0.195 | 0.167 | 0.177 |
| 19 | *Rhabdophis nuchalis* | 0.201 | 0.180 | 0.204 | 0.211 | 0.203 | 0.213 | 0.209 | 0.209 | 0.202 | 0.164 | 0.174 | 0.178 |
| 20 | *Hebius craspedogaster* | 0.207 | 0.193 | 0.227 | 0.201 | 0.223 | 0.209 | 0.214 | 0.199 | 0.199 | 0.181 | 0.180 | 0.172 |
| 21 | *Opisthotropis latouchii* | 0.207 | 0.187 | 0.222 | 0.212 | 0.198 | 0.187 | 0.209 | 0.202 | 0.193 | 0.161 | 0.174 | 0.166 |
| 22 | *Trimerodytes annularis* | 0.202 | 0.183 | 0.222 | 0.191 | 0.212 | 0.201 | 0.212 | 0.210 | 0.199 | 0.181 | 0.172 | 0.161 |
| 23 | *Trimerodytes percarinatus* | 0.210 | 0.180 | 0.210 | 0.200 | 0.198 | 0.207 | 0.196 | 0.196 | 0.192 | 0.196 | 0.169 | 0.169 |

**Table 1 Pairwise uncorrected genetic distances (*p*-distance) based on COI (continued)**

| Number | Species | 13 | 14 | 15 | 16 | 17 | 18 | 19 | 20 | 21 | 22 |
| --- | --- | --- | --- | --- | --- | --- | --- | --- | --- | --- | --- |
| 14 | *Lycodon rosozonatus* | 0.180 |  |  |  |  |  |  |  |  |  |
| 15 | *Lycodon rufozonatus* | 0.170 | 0.056 |  |  |  |  |  |  |  |  |
| 16 | *Sibynophis chinensis* | 0.193 | 0.176 | 0.169 |  |  |  |  |  |  |  |
| 17 | *Thermophis baileyi* | 0.181 | 0.176 | 0.174 | 0.180 |  |  |  |  |  |  |
| 18 | *Thermophis zhaoermii* | 0.181 | 0.178 | 0.181 | 0.186 | 0.093 |  |  |  |  |  |
| 19 | *Rhabdophis nuchalis* | 0.169 | 0.192 | 0.184 | 0.172 | 0.180 | 0.186 |  |  |  |  |
| 20 | *Hebius craspedogaster* | 0.180 | 0.191 | 0.183 | 0.184 | 0.178 | 0.186 | 0.161 |  |  |  |
| 21 | *Opisthotropis latouchii* | 0.187 | 0.167 | 0.175 | 0.175 | 0.167 | 0.172 | 0.177 | 0.166 |  |  |
| 22 | *Trimerodytes annularis* | 0.181 | 0.192 | 0.186 | 0.189 | 0.163 | 0.167 | 0.167 | 0.181 | 0.152 |  |
| 23 | *Trimerodytes percarinatus* | 0.183 | 0.178 | 0.196 | 0.190 | 0.143 | 0.157 | 0.166 | 0.181 | 0.160 | 0.114 |
