# Supplementary material for: How Host Phylogeny, Diet, and Habitat Affect Gut Microbial Diversity in Wild Snakes
Source: Ecol Evol. 2026 Jul 1;16(7):e73902. doi: 10.1002/ece3.73902 (PMC13322667; doi:10.1002/ece3.73902)
Supplement: Supplementary file 4 — Appendix S4: Description of the feeding habits of 23 species of wild snakes. [file ECE3-16-e73902-s003.docx]

**Appendix S4 Description of the feeding habits of 23 species of wild snakes**

| **Species** | **Family** | **Group** | **Dietary description** | **Reference(s)** |
| --- | --- | --- | --- | --- |
| *Eryx tataricus* | Boidae | Vertebrate | Main food: desert small rodents; Supplementary food: lizards and small birds | Zhao *et al*., 1998; Editorial Board of Vertebrate Fauna of Xinjiang, 1999; Zhao, 2006; Wang *et al*., 2021; China Wildlife Conservation Association, 2023 |
| *Boiga kraepelini* | Colubridae | Vertebrate | Main food: small birds (57.14%) and lizards (42.86%); Supplementary food: bird eggs, chicks, frogs/rodents (rarely) | Stejneger, 1902; Zhao *et al*., 1998; Zhao, 2006; Liu *et al*., 2012; Guo *et al*., 2024 |
| *Boiga multomaculata* | Colubridae | Vertebrate | Main food: small arboreal lizards, birds and bird eggs; Supplementary food: small rodents | Zhao *et al*., 1998; Zhao, 2006; Liu *et al*., 2012; Guo *et al*., 2024 |
| *Elaphe carinata* | Colubridae | Vertebrate | Main food: rodents, birds and bird eggs, frogs, lizards, snakes | Zhao *et al*., 1998; Guo *et al*., 2024 |
| *Euprepiophis mandarinus* | Colubridae | Vertebrate | Small rodents | Zhao *et al*., 1998; Wang *et al*., 2021; Guo *et al*., 2024 |
| *Lycodon rosozonatus* | Colubridae | Vertebrate | Main food: frogs; Supplementary food: other snakes, lizards/small rodents (rarely) | Zhao *et al*., 1998; Zhao, 2006; Liu *et al*., 2012; Wang *et al*., 2021 |
| *Lycodon rufozonatus* | Colubridae | Vertebrate | Broadly omnivorous. Main food: toads, frogs, and fish; Supplementary food: lizards, small snakes, rodents, young birds, eat snakes (when food is scarce) | Zhao *et al*., 1998; Zhao, 2006; Liu *et al*., 2012; Guo *et al*., 2024 |
| *Thermophis baileyi* | Dipsadidae | Vertebrate | Main food: plateau loach, highland frog | Zhao *et al*., 1998; Zhao, 2006; Liu *et al*., 2012 |
| *Thermophis zhaoermii* | Dipsadidae | Vertebrate | Main food: plateau loach, highland frog | Guo *et al*., 2008; Guo *et al*., 2024 |
| *Hebius craspedogaster* | Natricidae | Vertebrate | Main food: small freshwater fish and loaches; Supplementary food: frogs, toads, tadpoles and aquatic insects (rarely) | Hu *et al*., 1987; Zhao *et al*., 1998; Wang *et al*., 2021; Guo *et al*., 2024 |
| *Opisthotropis latouchii* | Natricidae | Invertebrate | Main food: earthworms (>80%); Supplementary food: small freshwater fish, tadpoles, aquatic shrimps, aquatic insect larvae (rarely) | Boulenger, 1899; Zhao *et al*., 1998; Zhao, 2006; Liu *et al*., 2012; Wang *et al*., 2021 |
| *Rhabdophis nuchalis* | Natricidae | Invertebrate | Main food: earthworms; Supplementary food: firefly larvae and slugs (rarely) | Zhao *et al*., 1998; Zhao, 2006; Liu *et al*., 2012; Yoshida, 2020; Wang *et al*., 2021; Guo *et al*., 2024 |
| *Trimerodytes annularis* | Natricidae | Vertebrate | Fish and frog | Zhao *et al*., 1998; Zhao, 2006 |
| *Trimerodytes percarinatus* | Natricidae | Vertebrate | Fish, frog and tadpole | Zhao *et al*., 1998; Guo *et al*., 2024 |
| *Pareas hamptoni* | Pareidae | Invertebrate | Snail and slug | Boulenger, 1903; Zhao *et al*., 1998; Zhao, 2006; Wang *et al*., 2021 |
| *Pareas margaritophorus* | Pareidae | Invertebrate | Main food: terrestrial mollusks (snails and slugs) | Boulenger, 1899; Zhao *et al*., 1998; Zhao, 2006; Liu *et al*., 2012; Wang *et al*., 2021 |
| *Sibynophis chinensis* | Sibynophiidae | Vertebrate | Main food: lizards; Supplementary food: small snakes and frogs | Zhao *et al*., 1998; Wang *et al*., 2021; Guo *et al*., 2024 |
| *Deinagkistrodon acutus* | Viperidae | Vertebrate | Main food: rodents and frogs; Supplementary food: birds, lizards and toads | Zhao *et al*., 1998; Guo *et al*., 2022 |
| *Gloydius angusticeps* | Viperidae | Vertebrate | Main food: small rodents, plateau lizards and frogs; Supplementary food: insects, spiders and the young of pika (rarely) | Shi *et al*., 2018; Guo *et al*., 2022, 2024 |
| *Viridovipera stejnegeri* | Viperidae | Vertebrate | Main food: frogs, rodents and lizards | Zhao *et al*., 1998; Guo *et al*., 2022, 2024 |
| *Viridovipera yunnanensis* | Viperidae | Vertebrate | Main food: frogs, rodents and lizards | Zhao *et al*., 1998; Guo *et al*., 2022, 2024 |
| *Achalinus spinalis* | Xenodermidae | Invertebrate | Main food: earthworms ( > 95%) | Peters, 1869; Hu *et al*., 1987; Zhao *et al*., 1998; Zhao, 2006; Liu *et al*., 2012; Guo *et al*., 2024 |
| *Xenopeltis hainanensis* | Xenopeltidae | / | Frogs, rodents and earthworms | Zhao *et al*., 1998; Zhao, 2006 |

Boulenger GA. Catalogue of the Snakes in the British Museum (Natural History)[M]. Vol. III. London: Taylor and Francis,1899.

Boulenger GA. Descriptions of new snakes from Burma[J]. Annals and Magazine of Natural History, 1903, (7): 445-448.

Boulenger GA. On a collection of reptiles and batrachians made by Mr. J. D. La Touche in N.W. Fokien, China[J]. Proceedings of the Zoological Society of London, 1899, 159-172.

China Wildlife Conservation Association. A Guide to the Key Protected Wildlife of China (Vol.2: Mammals and Reptiles)[M]. Fuzhou: Strait Publishing House, 2023.

Editorial Board of Vertebrate Fauna of Xinjiang. Vertebrate Fauna of Xinjiang (Reptilia)[M]. Urumqi: Xinjiang Science and Technology Health Press, 1999, 96-97.

Guo P, Che J. Snakes in Qinghai-Xizang plateau[M]. Beijing: Science Press, 2024.

Guo P, Liu Q, Wu YY, *et al*. Pitvipers of China[M]. Beijing: Science Press, 2022.

Guo P, Liu S, Feng Z, *et al*. A new species of Thermophis (Serpentes: Colubridae) from western Sichuan, China[J]. Zootaxa, 2008, 1757: 57-68.

Hu SQ, Zhao EM, Liu CZ, *et al*. Atlas of Chinese Animals: Amphibians and Reptiles (2nd ed.)[M]. Beijing: Science Press, 1987.

Liu Y, Ding L, Lei J, *et al*. Eye size variation reflects habitat and daily activity patterns in colubrid snakes[J]. Journal of Morphology, 2012, 273(8):883-93.

Peters WCH. Über neue Gattungen und neue oder weniger bekannte Arten von Amphibien (Eremias, Dicrodon, Euprepes, Lygosoma, Typhlops, Eryx, Rhynchonyx, Elapomorphus, Achalinus, Coronella, Dromicus, Xenopholis, Anoplodipsas, Spilotes, Tropidonotus)[J]. Monthly Reports of the Royal Prussian Academy of Sciences in Berlin, 1869, 432-447.

Shi J, Yang D, Zhang W, *et al*. A New Species of the *Gloydius strauchi* Complex (Crotalinae: Viperidae: Serpentes) from Qinghai, Sichuan, and Gansu, China[J]. Russian Journal of Herpetology, 2018, 25(2): 126-138.

Stejneger L. A new opisthoglyph snake from Formosa[J]. Proceedings of the Biological Society of Washington, 1902, 15:15-17

Wang YZ, Cai B, Li JT, *et al*. China’s Red List of Biodiversity: Vertebrates (Vol. Ⅲ): Reptiles[M]. Beijing: Science Press, 2021.

Yoshida T, Ujiie R, Savitzky AH, *et al*. Dramatic dietary shift maintains sequestered toxins in chemically defended snakes[J]. PNAS, 2020, 117(11): 5964-5970.

Zhao EM, Huang MH, Zong Y, *et al*. Fauna Sinica: Reptilia, Squamata, Serpentes[M]. Beijing: Science Press, 1998.

Zhao EM. Snakes of China (Volume I & II)[M]. Hefei: Anhui Science and Technology Press, 2006.
